# BAFF neutralization impairs the autoantibody-mediated clearance of dead adipocytes and aggravates obesity-induced insulin resistance

**DOI:** 10.3389/fimmu.2024.1436900

**Published:** 2024-08-09

**Authors:** Melissa D. Lempicki, Jake A. Gray, Gabriel Abuna, Ramiro M. Murata, Senad Divanovic, Coleen A. McNamara, Akshaya K. Meher

**Affiliations:** ^1^ Department of Microbiology and Immunology, Brody School of Medicine, East Carolina University, Greenville, NC, United States; ^2^ School of Dental Medicine, East Carolina University, Greenville, NC, United States; ^3^ Department of Pediatrics University of Cincinnati College of Medicine, Cincinnati, OH, United States; ^4^ Division of Immunobiology, Cincinnati Children’s Hospital Medical Center, Cincinnati, OH, United States; ^5^ Center for Inflammation and Tolerance, Cincinnati Children’s Hospital Medical Center, Cincinnati, OH, United States; ^6^ Cardiovascular Research Center, Cardiovascular Division, Department of Medicine, University of Virginia, Charlottesville, VA, United States

**Keywords:** BAFF, B2 cell, IgG autoantibodies, obesity-induced insulin resistance, white adipose tissue, inflammation

## Abstract

B cell-activating factor (BAFF) is a critical TNF-family cytokine that regulates homeostasis and peripheral tolerance of B2 cells. BAFF overproduction promotes autoantibody generation and autoimmune diseases. During obesity, BAFF is predominantly produced by white adipose tissue (WAT), and IgG autoantibodies against adipocytes are identified in the WAT of obese humans. However, it remains to be determined if the autoantibodies formed during obesity affect WAT remodeling and systemic insulin resistance. Here, we show that IgG autoantibodies are generated in high-fat diet (HFD)-induced obese mice that bind to apoptotic adipocytes and promote their phagocytosis by macrophages. Next, using murine models of obesity in which the gonadal WAT undergoes remodeling, we found that BAFF neutralization depleted IgG autoantibodies, increased the number of dead adipocytes, and exacerbated WAT inflammation and insulin resistance. RNA sequencing of the stromal vascular fraction from the WAT revealed decreased expression of immunoglobulin light-chain and heavy-chain variable genes suggesting a decreased repertoire of B cells after BAFF neutralization. Further, the B cell activation and the phagocytosis pathways were impaired in the WAT of BAFF-neutralized mice. *In vitro*, plasma IgG fractions from BAFF-neutralized mice reduced the phagocytic clearance of apoptotic adipocytes. Altogether, our study suggests that IgG autoantibodies developed during obesity, at least in part, dampens exacerbated WAT inflammation and systemic insulin resistance.

## 1 Introduction

Expansion of the white adipose tissue (WAT) is the primary characteristic of obesity. Expanded visceral WAT exhibits adipocyte hypertrophy, adipocyte dysfunction, and death, accumulation of leukocytes, and low-grade inflammation. Such abnormalities in visceral WAT promote systemic glucose dysmetabolism (1). In murine models of high-fat diet (HFD)-induced obesity and insulin resistance (IR), B cells, T cells, and macrophages infiltrate gonadal WAT after as few as 4 weeks of HFD feeding and orchestrate WAT inflammation (2). Macrophages, the most abundant leukocyte (40-60%) in obese murine gonadal WAT, are key players in WAT inflammation and IR during obesity (3, 4). In both obese humans and murine models, macrophages congregate around dying adipocytes forming crown-like structures (CLS) (5), and the number of CLS is positively associated with WAT inflammation and systemic IR (6). Various mechanisms are proposed for the death of the adipocytes, such as necrosis, apoptosis, and necroptosis (5), and various mechanisms for the clearance of dead adipocytes by CLS macrophages, such as exophagy (7) and efferocytosis (8). Irrespective of the mechanisms, the clearance of dead adipocytes promotes healthy WAT remodeling and recovery from IR in HFD-induced obese mice (6).

C57BL/6J mice are the most popular model of murine obesity and IR. Strissel et al. have reported that continuous feeding of HFD to male C57BL/6J mice for 16 weeks results in the death of ~80% of adipocytes in the gonadal WAT and a significant increase in IR (6). However, after 20 weeks on HFD, the WAT inflammation was partly resolved with the removal of dead adipocytes and decreased IR compared to the 16-week HFD-fed mice. Calorie restriction in HFD-induced obese C57BL/6J mice also promotes healthy WAT remodeling with an initial increase in the number of CLS (9) and an increase in the number of specialized phagocytic macrophages (10), followed by a decrease in macrophage content and a decrease in systemic IR. These findings suggest a healthy remodeling of WAT by removal of dead adipocytes is beneficial for reducing IR.

Unhealthy WAT remodeling, that is, extensive death of adipocytes during obesity leads to a significant change in the adipocytokine profile that promotes IR (11). One such adipocytokine highly synthesized by WAT during obesity is the B cell-activating factor (BAFF, BLYS, or TALL-1) (12). BAFF belongs to the tumor necrosis factor (TNF) family of cytokines and is critical for the survival and differentiation of B2 cells and BAFF deficiency results in a complete arrest of B2 cell maturation past the transitional 1 stage (13). Antigen binding to B cell receptor on B2 cells induces a death signal which must be counteracted by BAFF signaling for the survival of antigen-stimulated B2 cells (14). In the absence of BAFF, antigen-stimulated B2 cells do not survive. Normally, B cell antigen receptors that recognize autoantigens are removed by the central, followed by the peripheral immunological tolerance mechanisms allowing the survival of B2 cells that do not recognize or remain anergic to autoantigens. However, high BAFF levels break the peripheral tolerance of B2 cells allowing the generation of autoantibody (autoAb)-producing autoreactive B2 cells as found in BAFF-overexpressing mice (15). The interaction of antigen-specific B cells with antigen-specific T cells is crucial for the production of isotype-switched antibodies (Abs). However, excess BAFF can drive the production of isotype-switched IgG autoAbs even without the help of T cells (16, 17).

Belimumab, a BAFF-neutralizing Ab approved by the U.S. Food and Drug Administration for the treatment of systemic lupus erythematosus (SLE) (18), decreases autoAb levels and disease severity (19). Harmful effects of IgG autoAbs occur *via* multiple mechanisms and are well-studied (20). Interestingly, IgG autoAbs generated in response to nerve injury clear myelin debris and promote axonal growth supporting a beneficial role of autoAbs (21). Winer et al. demonstrated the presence of IgG autoAbs against intracellular proteins normally expressed in multiple tissues and the serum of obese and IR individuals (2). Frasca et al., later reported that the plasma of obese individuals is rich in IgG autoAbs targeting intracellular proteins of adipocytes (22). Interestingly, IgG deposition around dead adipocytes is found in the CLSs of obese mice (2). While macrophages can clear dead adipocytes by exophagy and efferocytosis, it is unknown if adipocyte-specific autoAbs are generated in C57BL/6J mice, the widely studied murine models of diet-induced obesity and insulin resistance. It is also unknown if the autoAbs are deposited on the surface of dead adipocytes and promote their clearance by macrophages, thereby affecting WAT remodeling and systemic IR.

Here, we show that HFD-induced obesity resulted in the generation of IgG autoAbs that bind to apoptotic 3T3-L1 adipocytes. *In vitro*, IgG-rich plasma fraction from the obese mice engulfed more apoptotic adipocytes compared to the IgG-rich plasma from the lean mice. To induce healthy adipose tissue remodeling, we utilized two models. First, the long-term or 22-week HFD feeding of C57BL/6J mice as described by Strissel et al. (6) Second, a diet intervention model in which mice on an HFD for 12 weeks were then switched to a normal chow diet until week 18. In both models, BAFF neutralization significantly depleted the levels of IgG autoAbs, however, the BAFF-neutralized mice were more insulin resistant compared to the control mice. Extensive cellular phenotyping in the diet-intervention model revealed that BAFF neutralization effectively depleted the mature B2 cell subtypes in lymphoid organs and the stromal vascular fraction of gonadal WAT. Furthermore, the gonadal WAT of BAFF-neutralized mice had an increased number of dead adipocytes and increased inflammation. Bulk RNA sequencing of the stromal vascular fraction revealed a decreased repertoire of B cells, impaired B cell receptor signaling, immunoglobulin production, and phagocytosis pathways in the BAFF-neutralized mice. In this line, IgG-rich plasma fraction from BAFF-neutralized mice showed decreased engulfment of apoptotic adipocyte compared to the control mice plasma fraction. Altogether, our study suggests a beneficial role of BAFF and a novel role of autoAbs generated during obesity in the healthy remodeling of gonadal WAT and in the regulation of systemic IR.

## 2 Materials and methods

### 2.1 Mice

Eight or sixteen-week–old male C57BL/6J mice (#380050) were obtained from The Jackson Laboratory (Bar Harbor, ME). Mice were given water and fed a high-fat diet (Bio-Serv #F3282; 60% of calories from fat). Diet intervention mice were fed a high-fat diet for 12 weeks and then switched to a normal chow diet (Prolab IsoPro RMH 3000 5P76, LabDiet) for 6 weeks. The mice were injected every 2 weeks with 2 mg/kg body weight of the control Ab (IgG1k, BioX Cell, Lebanon, NH; catalog number BE0083) or anti-BAFF Ab (IgG1k, AdipoGen Life Sciences, San Diego, CA; catalog number AG-20B-0063PF). Body composition was determined using EchoMRI-700. All protocols involving animals were approved by the East Carolina University Animal Care and Use Committee and performed in an AAALAC-accredited facility in accordance with current NIH guidelines.

### 2.2 Metabolic studies

The glucose tolerance test (GTT) and insulin tolerance test (ITT) were performed as described earlier (23). Briefly, for the GTT, mice were fasted overnight with access to water and then injected intraperitoneally with glucose (1 g/kg body weight). Blood glucose levels were monitored kinetically for 2 hours by tail clipping and testing a drop of blood using a Contour Next One glucometer. For the ITT, mice were fasted for 4 hours and then injected intraperitoneally with 2 units/kg body weight of insulin (Humulin R, Eli Lilly and Company, Indianapolis, IN, USA). Blood glucose levels were monitored as described for GTT.

### 2.3 Isolation of SVF from adipose tissue

The left and the right gonadal white adipose tissue (WAT) was excised from the mouse and one of the fat pads was used for the isolation of the stromal vascular fraction (SVF). The tissue was minced with scissors and digested for 1 hour in Hanks Balanced Salt Solution (Gibco) with 2% bovine serum albumin (BSA) and 2 mg/ml of collagenase type I shaking at 37°C. After digestion, tissue was filtered through a 500 μM nylon mesh strainer and washed with HBSS with 2% BSA. The cells were centrifuged (450xg for 5 min) to allow the SVF to separate with the adipocytes floating on the top of the buffer. The SVF pellet was collected, and red blood cells were lysed. Cells were washed, centrifuged, and resuspended in various buffers depending on downstream use.

### 2.4 Flow cytometry quantification

Cell preparation for flow cytometry was performed as previously described (24). After the collection of the spleen, blood, and peritoneal lavage samples, the spleen was passed through a 70 μm pore size strainer, and red blood cells were lysed from splenocytes, 50 to 100 μL of blood, and peritoneal cells. The SVF was isolated as described above. The cells were blocked with purified anti-mouse CD16/32 (BioLegend, San Diego, CA; #101302) and stained with the following fluorescent conjugated antibodies from BioLegends and FisherScientific for 30 minutes on ice: PE/Dazzle 594 anti-CD19 (#115554), APC/Fire 810 anti-B220 (#103278), APC/Fire 750 anti-CD21 (#123434), Brilliant Violet 510 anti-CD23 (#101623), Spark Blue 550 anti-major histocompatibility complex II (#107662), Brilliant Ultraviolet 395 anti-IgD (#564274), Brilliant Violet 786 anti-CD38 (#740887), PE anti-IgM (#406508), Brilliant Violet 421 anti-CD138 (#142508), Brilliant Ultraviolet 661 anti-CD3 (#741562), PE Cyanine7 anti-F4/80 (#25-4801-82), PerCP-Cy5.5 anti-GL-7 (#144610), Brilliant Ultraviolet 805 anti-CD4 (#612900), Super Bright 436 anti-Gr1 (#62-9668-82), Brilliant Violet 480 anti-CD8 (#566096), Brilliant Violet 605 anti-CD86 (#105037), Brilliant Violet 650 anti-Ly6C (#105037), Brilliant Violet 711 anti-CD206 (#141727), Brilliant Blue 515 anti-CD45 (#564590), PerCp anti-CD117 (#105822), PE-Cy7 anti-NK-1.1 (#108714), APC-R700 anti-CD5 (#565505), Brilliant Violet 650 anti-CD11c (#117339), Alexa Fluor 488 CD170 (#155524), and APC anti-CD11b (#17-0112-83). Invitrogen DAPI (4’,6-diamidino-2-phenylindole, dihydrochloride) (#D1306) was used for staining the dead cell. The samples were run on the flow cytometer machine Cytek Biosciences Cytek Aurora, USA. Data analysis and quantification were performed using FlowJo v10.6.2.

### 2.5 Liver triglyceride and Alanine transaminase, and plasma lipid quantification

Liver triglyceride levels were quantified from frozen liver using a Triglyceride Colorimetric Assay Kit (Cayman Chemicals; #10010303) per manufacturer’s instructions. Alanine transaminase (ALT) levels were quantified from plasma using the Alanine Transaminase Colorimetric Activity Assay Kit (Cayman Chemicals; #700260) according to the manufacturer’s protocol. Plasma triglyceride, non-esterified fatty acids, and cholesterol levels were measured by the Mouse Metabolic Phenotyping Center at the University of Cincinnati Medical Center.

### 2.6 Plasma cytokine quantification

Plasma cytokine levels were measured using the Milliplex MAP Mouse Cytokine/Chemokine Magnetic Bead Panel – Immunology Multiplex Assay (Millipore, #MCYTOMAG-70K) according to the manufacturer’s protocol using Invitrogen Luminex™ 200™ Instrument System.

### 2.7 Immunohistochemistry

Five micrometer gonadal WAT cross-sections were hydrated with HistoClear Clearing Agent (MilliporeSigma), 100% ethanol, 90% ethanol, 70% ethanol, 20% ethanol, and then water. Antigen retrieval was performed by boiling the slides in the Antigen Unmasking Solution (Vector Laboratories, Burlingame, CA; #H-3300). After antigen retrieval, the tissue sections were treated with 10% donkey serum and incubated with primary Ab for 14 to 16 hours at 4°C. Primary Abs were detected by fluorescent secondary antibodies. The slides were mounted with ProLong Gold Antifade Mountant with DAPI (Invitrogen, #P36931) before image acquisition on a Laxco LMI 6000 Series Inverted microscope (Fisher Scientific). The primary antibodies used were anti-FABP4/A-FABP (Novus Biologics; #AF1443), anti-BAFF (Invitrogen; #MA5-29617), anti-Mac-2 (Cederlane; #CL8942AP), anti-phospho MLKL Ser358 (Thermo Fisher; #PA5-105678), anti-IgG2a/2b (BD Biosciences; #553397), and anti-IgM (BD Biosciences; #553435). The secondary antibodies used are Donkey anti-Goat Alexa Fluor Plus 488 (ThermoFisher; #A32814), Donkey anti-Rabbit Alexa Fluor Plus 555 (ThermoFisher; #A32794), and Donkey anti-Rat Alexa Fluor Plus 647 (ThermoFisher; A48272).

The area of adipocytes was calculated using ImageJ software version 1.53 (NIH, Bethesda, MD; http://imagej.nih.gov/ij). The number of crown-like structures per image was counted manually. The authors quantifying the images were blinded for the treatment strategy.

### 2.8 Western blot

Gonadal white adipose tissue was isolated from mice on HFD for 11 weeks and age-matched NCD controls. Adipose tissue was homogenized in a bead mill with TPER buffer (ThermoFisher Scientific #78510) containing a protease inhibitor cocktail (Sigma-Aldrich). The extracted protein was analyzed using 4-12% Tris-Glycine SDS-PAGE. The primary antibodies used are IgG antibody (Abcam, #ab6709) and GAPDH antibody (Cell Signaling Technology, #cs2118), and the IRDye-conjugated secondary antibodies were from LI-COR, and the blots were detected, and band intensities were quantified using LI-COR’s Image Studio software version 5.2.5.

### 2.9 RNA sequencing and data analysis

The stromal vascular fraction was isolated as described above and sent to SingulOmics (Bronx, New York) for bulk RNA sequencing (paired-end) using NovaSeq 6000. FPKM (expected number of Fragments Per Kilobase of transcript sequence per Million base pairs sequenced) was used for estimating gene expression levels. Using the FPKM values, log2 fold change (control Ab-treated SVF vs anti-BAFF Ab-treated SVF) values were calculated. Genes with a p-adjusted value below 0.05 were considered significantly regulated. For pathway analysis, significantly downregulated genes were analyzed by Gene Ontology analysis using the Gene Ontology Consortium website.

### 2.10 Plasma immunoglobulin and autoantibody quantification

IgG2b, IgG3, IgA, and IgM immunoglobulins were quantified from mouse plasma and 10 μg of homogenized gonadal WAT using Ig Isotyping Mouse Instant ELISA Kit (Thermo Fisher Scientific, #88-50660-22). Autoantibodies were quantified from the plasma by GeneCopiea (Rockville, MD).

### 2.11 Preparation of bone-marrow-derived macrophages and differentiation of 3T3-L1 adipocytes

The bone-marrow-derived macrophages and 3T3-L1 adipocytes were prepared as described before (23). Briefly, bone marrow cells from the femur and tibia of WT C57BL6/J mice were collected, red blood cells were lysed with lysis buffer and the cells were cultured at a density of 1 to 2×10^6^ cells/mL in RPMI 1640 medium containing 10% heat-inactivated fetal bovine serum (HI FBS) and 1× antibiotic-antimycotic, supplemented with 20% L929 (NCTC clone 929; ATCC, Manassas, VA) culture supernatant. The culture medium was changed on the fourth day and then on every second day.

3T3-L1 fibroblasts were cultured in DMEM supplemented with 10% HI FBS at 37°C, 95% relative humidity, and 5% CO2. For differentiation into adipocytes, cells were grown to confluence and treated for four days with differentiation media containing DMEM, 10% HI FBS, 1x antibiotic-antimycotic, 0.25 U/mL insulin, 0.5 mM 3-isobutyl-1-methylxanthine, 0.025 mM dexamethasone, followed by treatment for two days with post-differentiation media containing DMEM, 10% HI FBS, 0.25 U/mL insulin and then adipocytes were maintained in DMEM with 10% heat-inactivated FBS.

### 2.12 Apoptosis of 3T3-L1 adipocytes

Apotosis was induced in fully differentiated 3T3-L1 adipocytes as described earlier (25). Briefly, the adipocytes were washed with phosphate-buffered saline and subjected to UV radiation at 600 mJ/cm^2^ using Stratalinker 1800. 3T3-L1 adipocytes were then incubated overnight in DMEM without HI FBS. YOPRO (1 μM) was used to confirm the induction of apoptosis using ThermoFisher Scientific EVOS epifluorescent microscope.

### 2.13 Identification of autoantibodies to apoptotic adipocytes

10 μl of plasma from mice on normal chow diet (NCD) or high-fat diet (HFD) was added to healthy and apoptotic 3T3-L1 adipocytes. Cells were incubated for 1 hour on ice and then washed with phosphate-buffered saline. Cells were then stained with YOPRO (1 μM), Hoechst (1 μg/mL), and Dylight anti-mouse IgG and imaged with a ThermoFisher Scientific EVOS epifluorescent microscope. Alternatively, apoptotic cells were incubated with 10 μl of plasma from control antibody and anti-BAFF antibody-treated diet intervention mice.

### 2.14 Fractionation of plasma to produce IgG-rich plasma

To produce the IgG-rich plasma 300 μL of plasma from NCD, HFD, control antibody-treated, or anti-BAFF antibody-treated mice was filtered with 0.2 μm filter, fractionated using the size exclusion chromatography column Superose 6 Increase 10/300 GL column connected to ACTA pure 25 chromatography system. The proteins were eluted as 1 ml fractions in phosphate-buffered saline. Based on molecular weight corresponding to 155 kDa, fractions 17 and 18 were predicted to contain the IgG which was confirmed by analyzing the fractions on a 12% Tris-Glycine SDS-PAGE under reducing conditions (with 2-Mercaptoethanol). The two 1 mL fractions were then concentrated down to 300 μL, to maintain the original concentration of plasma IgG.

### 2.15 Phagocytosis assay

Macrophages were stained with DiI (1:1000, #V-22885; Molecular Probes) and seeded at a density of 500,000 cells per well in a 4-well chamber slide in complete RPMI media and allowed to attach overnight. Apoptosis was induced in 3T3-L1 adipocytes as described above. Apoptotic adipocytes were stained with BODIPY™ 493/503 (1 ug/mL, #D3922; ThermoFisher). The phagocytosis assay began with the addition of 2 million apoptotic adipocytes which were incubated for 0 and 6 hours at 37°C. Phagocytosis was determined by fluorescent imaging with EVOS epifluorescent microscope.

Alternatively, macrophages were stained with CellTrace™ Violet (5 μM; Thermo Fisher Scientific #C34571) and seeded at a density of 200,000 cells per well in a 24-well flat bottom treated tissue culture plate and allowed to attach overnight. Apoptotic adipocytes were stained with BODIPY™ 493/503 (1 ug/mL; Thermo Fisher Scientific #D3922), DiI (1:1000; Molecular Probes, #V-22885), and CypHer5E NHS Ester (1 μM; Cytiva, # PA15401). 400,000 apoptotic adipocytes were added to the macrophages with 25 μL of IgG-rich plasma from NCD or HFD mice and IgG-rich plasma from control antibody-treated or anti-BAFF antibody-treated diet intervention mice. Cells were incubated together for 6 hours at 37°C. After incubation, cells were washed, trypsinized, and phagocytosis was analyzed by flow cytometer. Macrophages were treated with cytochalasin D (10 μmol/L; Cayman Chemicals) for 1 hour as a negative control for the phagocytosis assay.

### 2.16 Seahorse assays to determine the metabolic activity of adipocytes

The 3T3-L1 fibroblasts were seeded on XFe24 well microplates coated with Poly-L-lysine solution (P4832, Sigma-Aldrich, USA) and differentiated into adipocytes as described earlier. A mitochondrial stress test on Seahorse XFe24 extracellular flux analyzer was performed using a Seahorse XFe24 Extracellular Flux Analyzer as described previously (26). Concentrations of the drugs injected are: 1st injection Oligomycin A (10 μM, Sigma-Aldrich, #75351), 2nd injection BAM15 (10 μM, Cayman Chemicals, #17811), and the 3rd injection Antimycin A (100 μM, Sigma-Aldrich, #A8674) + Rotenone (10 μM, Sigma-Aldrich, #557368). Oxygen consumption rate (OCR) and extracellular acidification rate (ECAR) were measured four times before the injection of Oligomycin A and after each injection.

### 2.17 Statistical analysis

The data were analyzed by using GraphPad Prism 8 (GraphPad Software, La Jolla, CA) and Excel (Microsoft Corporation, Redmond, WA), and they are presented as means ± SEM. Differences between the mean values of the two groups were determined by using t-tests. The means of multiple groups were compared by using a one-way analysis of variance. The D’Agostino-Pearson normality test was performed on each group. If the P value was not significant (>0.05), a two-tailed parametric test was used; if the P value was significant (<0.05) or if the number of samples was four, a two-tailed nonparametric t-test (U-test) was used to determine significant differences between the groups. In the multiple comparisons, if a significant difference was found among the groups, pairs of groups were compared by using a parametric or nonparametric t-test. The number of crown-like structures in gonadal WAT between the groups were compared using a generalized estimating equation using R environment. Statistical analyses are provided in each figure legend. Differences between the groups were considered significant when P < 0.05. P values >0.05 are indicated in the graphs.

## 3 Results

### 3.1 IgGs from obese mice promote the phagocytosis of apoptotic adipocytes *in vitro*

**Figure 1:**
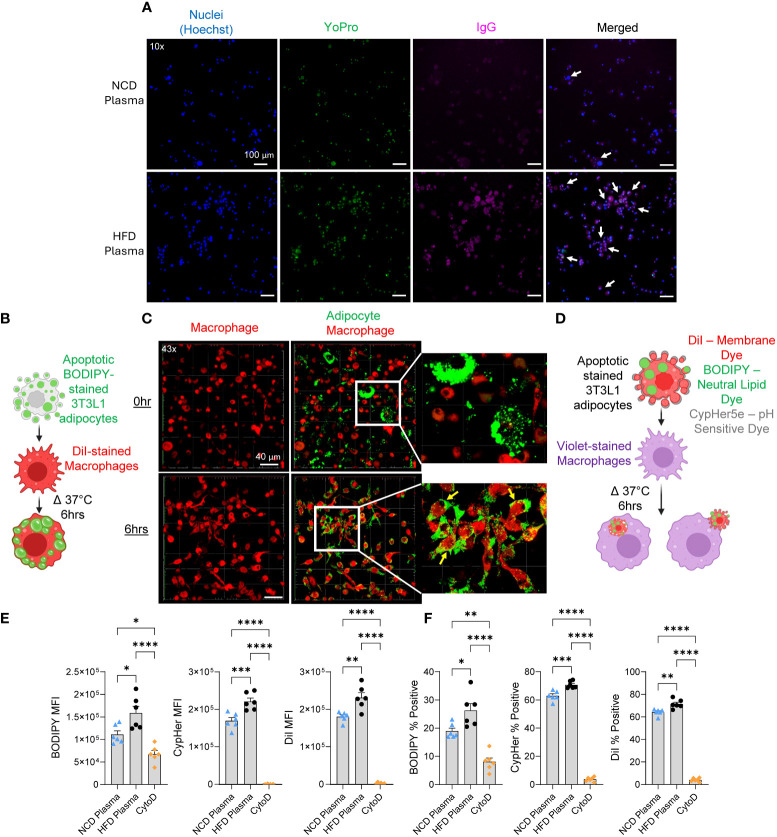
Autoantibodies from obese mice promote phagocytosis in an *in vitro* model. **(A)** Representative fluorescent images of apoptotic 3T3-L1 adipocytes incubated with plasma from NCD or HFD-fed mice stained for nucleus (Hoechst-blue), apoptosis (YoPro-green), and IgG (pink, anti-IgG antibody). White arrows indicate cells positive, for Hoechst, YoPro, and IgG. **(B)** Schematic of phagocytosis experiment used for confocal imaging. **(C)** 3D confocal images of macrophages (DiI-red) co-cultured with apoptotic 3T3-L1 adipocytes (BODIPY-green) for 0 hours (0hr) and 6 hours (6hr). Parts of the images were digitally enlarged to show the localization of BODIPY stains inside the macrophages with yellow arrows. **(D)** Schematic of phagocytosis experiment used for flow cytometry. In this experiment, macrophages were stained with CellTrace Violet, and adipocytes were stained with three dyes: BODIPY, CypHer5E, and DiI. **(E, F)** Flow cytometry of macrophages after co-cultured with apoptotic adipocytes in the presence of IgG-rich plasma fraction from NCD or HFD-fed mice. **(E)** The amount of apoptotic cell uptake was assessed by median fluorescent intensity (MFI) of BODIPY, CypHer5e, and DiI of macrophages. **(F)** The percentage of phagocytic macrophages was determined by the percent of BODIPY, CypHer5e, and DiI-positive macrophages. Values are expressed as means + SEM. *, p<0.05; **, p<0.01; ***, p<0.001 ****, p<0.0001 by parametric unpaired t-test. n=3 wells per treatment, 3 images each **(A)**, n=1 well per treatment, 10 images each **(C)**, n=6 **(E, F)**. Scale bars: 100 μm **(A)**; 40 μm **(C)**.

In obese and IR humans, B cells in the fat tissues secrete IgG autoAbs specific for apoptotic adipocytes (22), however, it is not clearly understood if they are formed in diet-induced obese mice. We first determined IgG levels in the gonadal WAT of HFD-fed (for 11 weeks) obese C57BL/6J mice, and age-matched normal chow diet (NCD)-fed mice and found no significant differences (**Supplementary Figure 1A**) suggesting that reports of increased IgG deposition around dead adipocytes (2) may be due to increased IgG autoAbs. Next, we exposed murine 3T3-L1 adipocytes to UV radiation and confirmed apoptosis by uptake of YO-PRO stain (25) (**Supplementary Figure 1B**). We then incubated the healthy or apoptotic 3T3-L1 adipocytes with plasma from mice on NCD or HFD (11 weeks) and probed for IgG bound to the surface of the adipocytes. IgG binding was higher on the apoptotic adipocytes treated with HFD plasma compared to those treated with NCD plasma (**Figure 1A**) indicating that obese mice produce autoAbs against apoptotic adipocytes. As expected, the healthy adipocytes had little to no YO-PRO staining and no IgG binding (**Supplementary Figure 1C**), indicating that the autoAbs were specific to dead adipocytes.

AutoAbs are necessary for the recognition and removal of dead cells through antibody-dependent cellular phagocytosis (ADCP) (27). We examined if the increase in IgG autoAbs found in obese mice affected the rate of phagocytic removal of dead adipocytes. To do so we developed an *in vitro* phagocytosis assay utilizing apoptotic 3T3-L1 adipocytes. To confirm the efficacy of the method, apoptotic 3T3-L1 adipocytes were stained with BODIPY, a fluorescent neutral lipid dye, and incubated with murine bone-marrow-derived macrophages stained with DiI, a fluorescent membrane dye (**Figure 1B**). Phagocytosis was confirmed after 6 hours with confocal microscopy based on the uptake of BODIPY into the macrophages (**Figure 1C**). For quantitative analysis of phagocytosis, we developed a flow cytometry-based assay. Since IgM antibodies can also bind to dead cells and promote their clearance by macrophages (28), we fractionated the mouse plasma samples using size exclusion chromatography and pooled 155 kDa molecular weight fractions. Next, apoptotic 3T3-L1 adipocytes were stained with three fluorescent dyes, BODIPY, DiI, and CypHer5e (binds to cell surface proteins and is fluorescent in acidic pH) and incubated with the macrophages stained with CellTrace Violet in the presence of IgG-rich plasma fractions from NCD lean mice or HFD-fed (11 weeks) obese mice (**Figure 1D**). HFD IgG-rich plasma significantly increased the engulfment of apoptotic adipocytes stained with all three dyes (**Figure 1E** and **Supplementary Figure D**). The HFD IgG-rich plasma also significantly increased the percentage of macrophages positive for each dye (**Figure 1F**). Particularly, the percentages of macrophages that were positive for Dil or CypHer5e were higher than the percentages of BODIPY. This is likely due to all of the apoptotic bodies of the adipocytes may not contain lipid droplets (BODIPY+), however, all apoptotic bodies have membranes (DiI+) and proteins (CypHer5e+). As a negative control, cytochalasin D treatment was used, which significantly attenuated phagocytosis. Together these results show that autoAbs in obese C57BL/6J mice enhance phagocytic clearance of dead adipocytes *in vitro*.

### 3.2 BAFF neutralization impairs the partial gain in insulin sensitivity in a long-term high-fat diet model of obesity

**Figure 2:**
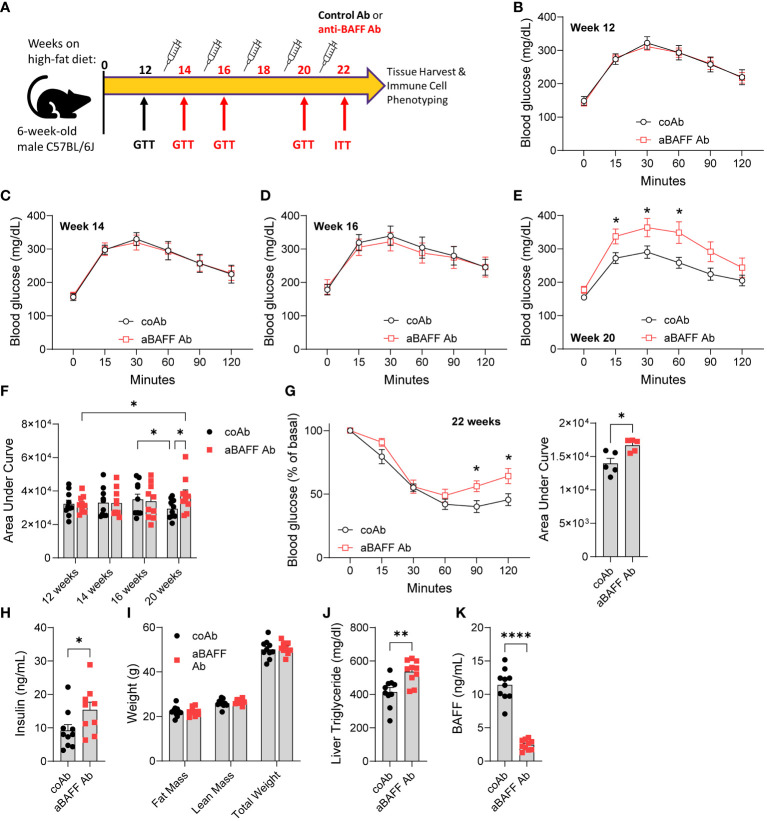
Anti-BAFF antibody-treated mice had increased insulin resistance in the long-term high-fat diet model. **(A)** Representative timeline of antibody treatment for mice on HFD. **(B)** GTT and of mice on HFD for 12 weeks. coAb: mice treated with a control antibody, and aBAFF Ab: mice treated with anti-BAFF antibody. **(C)** GTT at 14 weeks, **(D)** GTT at 16 weeks, and, **(E)** GTT at 20 weeks on HFD. **(F)** Area under the curve (AUC) of GTTs from weeks 12, 14, 16, and 20. **(G)** ITT and AUC at 22 weeks on HFD. **(H)** Plasma insulin levels in ng/ml; **(I)** fat mass, lean mass, and total body weight of controls and anti-BAFF Ab-treated mice were determined by EchoMRI; **(J)** liver triglyceride quantified by colorimetric assay; and **(K)** plasma BAFF levels in ng/ml after the completion of the experiment. Values are expressed as means + SEM. *, p<0.05; **, p<0.001; ****, p<0.0001 by parametric unpaired t-test. n=9-10 **(B-F, H-K)** and n=5 **(G)**.

BAFF neutralization results in depletion of mature B2 cell populations which eventually leads to reduced antibody levels (29). Strissel et al. reported that, in the long-term HFD feeding model, insulin resistance peaks at 16 weeks on HFD, and then partly decreases at week 20 as the gonadal WAT undergoes healthy remolding (6). To determine the effect of BAFF neutralization and antibody depletion, six weeks old male C57BL/6J mice were placed on an HFD for 12 weeks to develop advanced IR and experimental groups were determined based on a glucose tolerance test (GTT) (12 weeks, **Figures 2A, B**). Mice were then given an injection of 2 mg/kg of anti-BAFF Ab (Sandy-2) or an isotype control Ab every 2 weeks (dosage (30)) for a total of 10 weeks. To determine glucose intolerance, GTTs were used over the course of 10 weeks. As expected, in the control group, glucose intolerance remained high till week 16, however, there was no difference with the anti-BAFF Ab-treated mice (**Figures 2C, D, F)**. Therefore, in a small cohort, we confirmed the effect of BAFF neutralization by drawing a small amount of blood from the tail vein and determining the depletion of B2 cells by flow cytometry (**Supplementary Figure 2A**). Flow cytometry gating strategy is provided in **Supplementary Figure 3A**. At week 20, as expected, the control Ab-treated mice cleared glucose faster than the same group of mice at week 16 (**Figure 2F**). Interestingly, at week 20, glucose intolerance of the anti-BAFF Ab-treated mice was significantly higher than the control Ab-treated mice, and also higher than the anti-BAFF Ab group at 12 weeks (**Figures 2E, F**). To determine the development of insulin resistance, we performed an ITT on week 22 and found that the anti-BAFF Ab-treated mice were more insulin resistant compared to the control Ab-treated mice (**Figure 2G** **).** In this line, plasma insulin levels were higher in the anti-BAFF Ab-treated mice (**Figure 2H**). There were no differences between the fat mass, lean mass, total body weight (**Figure 2I**), gonadal WAT weight (**Supplementary Figure 2B**), or overall weight gain (**Supplementary Figure 2C**) between the groups suggesting that the differences in IR are independent of weight gain. BAFF neutralization also increased liver steatosis evidenced by increased liver triglyceride levels (**Figure 2J** **).** A BAFF ELISA on the plasma of these mice confirmed reduced BAFF levels in the anti-BAFF Ab-treated mice (**Figure 2K**).

Altogether, these results demonstrate that BAFF neutralization attenuated partial recovery of glucose dysmetabolism at 20 weeks after HFD and promoted liver steatosis in the C57BL/6J mice.

### 3.3 BAFF neutralization affected the dynamics of B cell subtypes and depleted circulating autoantibodies in the long-term high-fat diet model

Among the B cell subtypes, B1 cells are known to attenuate the severity of obesity-induced IR by producing IgM antibodies (29), whereas B2 cells exacerbate obesity-induced IR by IgG antibodies (2). Notably, IgGs isolated from the plasma of obese mice can promote IR in genetically B cell-deficient muMT mice only if the mice are obese, suggesting diet-induced obesity is crucial for the pathogenic activity of IgGs (2). Mechanistically, IgGs from obese mice bind to macrophages in the gonadal WAT *via* Fc receptors, inducing polarization of macrophages to M1 leading to the production of TNF-α, which promotes WAT inflammation and increases IR (2). On the other hand, B1 cells, more specifically, B1b cells, can migrate to the gonadal WAT of obese mice and secrete natural IgM to oxidation-specific epitopes and reduce the secretion of proinflammatory cytokines such as TNF-α and IFN-γ by M1 macrophages (31). Since the anti-BAFF Ab-treated mice showed increased glucose dysmetabolism than the control Ab-treated mice, we first determined the levels of immunoglobulins in the plasma of these mice. Unexpectedly, no significant difference between IgG isotypes or IgM was found, though lambda chain was significantly decreased (**Supplementary Figure 4A**). This questioned whether the anti-BAFF Ab treatment was effective enough to deplete mature B2 cells. Therefore, we performed a detailed analysis of B cell subtypes in the spleen, blood, peritoneal cavity, and the SVF of the gonadal WAT using flow cytometry. Apart from B cells, we also determined if the anti-BAFF Ab treatment affected the dynamics of other innate and adaptive immune cells such as CD4+ T cells, CD8+ T cells, M1 and M2 macrophage subtypes, natural killer (NK) cells, neutrophils and eosinophils (flow cytometry gating strategy in **Supplementary Figures 3A, B**).

We found that, in the spleens of anti-BAFF Ab-treated mice, the B2 cell subtypes past the transitional 1 stage were significantly depleted (**Supplementary Figure 4B**). B2 cell subtypes were also effectively depleted within the blood and peritoneal cavity of anti-BAFF Ab treated mice (**Supplementary Figures 4C, D**), thus confirming the activity of the anti-BAFF Ab. The B1a and B1b cell numbers were reduced in the spleen of anti-BAFF Ab-treated mice (**Supplementary Figure 4B**), however, their numbers were not affected in the blood and peritoneal cavity (**Supplementary Figures 4C, D**). Depletion of B2 cells did not affect the number of CD4+ T cells and CD8+ T cells in the spleen. Furthermore, in blood and peritoneal cavity, anti-BAFF Ab treatment depleted only B2 cells, but did not affect the number of B1a cells, B1b cells, CD4+ T cells, and CD8+ T cells (**Supplementary Figures 4C, D**). Interestingly, in the SVF of the gonadal WAT, B2 cells were not effectively depleted in the anti-BAFF Ab-treated mice (**Supplementary Figure 4E**). This could be due to decreased penetrance of anti-BAFF Ab in these overly obese mice or high levels of BAFF within the WAT. B1a cell, B1b cell, CD4+ T cell, and CD8+ T cell numbers were also similar between the groups in the SVF (**Supplementary Figure 4E**). We also did not find significant differences in the M1 and M2 macrophage populations, NK cells, and neutrophils in the SVF between the groups (**Supplementary Figure 4F**).

In this model, extensive death of adipocytes in the gonadal WAT occurs at week 16 of HFD, followed by the appearance of new and small adipocytes found at week 20 of HFD (6). Accumulation of dead adipocytes as identified by the increased number of CLSs, is a critical marker of impaired phagocytic removal of dead adipocytes (32). Therefore, we determined the number of CLSs per field size and the distribution of live adipocytes in the gonadal WAT of the anti-BAFF Ab and control Ab-treated mice. However, we did not find any significant difference in the number of CLS nor adipocyte size distribution (**Supplementary Figures 5A–C**), suggesting the gonadal WATs were equally remodeled by the end of the experiment, which was 23 weeks on HFD. Probably, the gonadal WAT phenotype, which impaired WAT remodeling leading to excess lipid spillover, is propagated to other compartments such as the liver (**Figure 2J**) at the time of investigation. Since the anti-BAFF Ab treatment did not affect innate and adaptive cell populations in the gonadal WAT and the circulating immunoglobulins, but increased IR and liver steatosis, we determined the levels of autoAbs in the plasma using an antigen array, which has 120 self-antigens (**Supplementary Table 1**). The results revealed the depletion of multiple IgG and IgM autoAbs in the plasma of the anti-BAFF Ab-treated mice (**Supplementary Figures 4G, H**), confirming the activity of the anti-BAFF Ab.

Altogether, these results suggest that BAFF neutralization in the long-term obesity model specifically depleted B2 cells in most body compartments except for the gonadal WAT and depleted circulating IgG and IgM autoAbs.

### 3.4 BAFF neutralization impairs improvements in insulin sensitivity in a high-fat to low-fat diet intervention model

**Figure 3:**
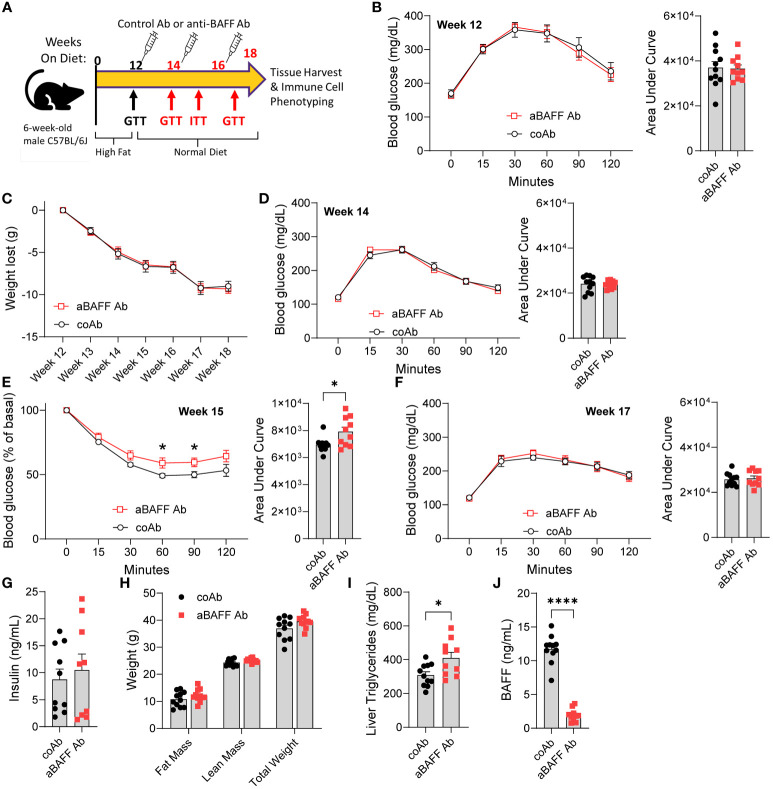
Anti-BAFF antibody treatment impairs recovery from insulin resistance in the diet intervention model. **(A)** Representative timeline of diet change and antibody treatment of mice. **(B)** GTT and area under the curve (AUC) of mice on HFD for 12 weeks. **(C)** Weight loss of control and anti-BAFF Ab-injected mice after switching from HFD to NCD. **(D)** GTT and AUC of mice on NCD for 2 weeks (week 14). **(E)** ITT and AUC of mice 3 weeks on NCD (week 15). **(F)** GTT and AUC of mice 5 weeks on NCD (week 17). **(G)** Plasma insulin levels in ng/ml; **(H)** fat mass, lean mass, and total body weight of controls and anti-BAFF Ab-treated mice were determined by EchoMRI; **(I)** liver triglyceride levels; and **(J)** Plasma BAFF levels in ng/ml after the completion of the experiment. Values are expressed as means + SEM. *, p<0.05; ****, p<0.0001 by a parametric unpaired t-test, n=10-11 **(B-F, H-J)** and n=4-5 **(G)**.

In the long-term HFD feeding model, the lack of B2 cell depletion within the gonadal WAT prompted us to use another model of healthy WAT remodeling. In this model, we introduced a diet intervention on obese mice and examined the effects of BAFF neutralization on IR (**Figure 3A**). Male C57BL/6J mice were placed on an HFD for 12 weeks to induce IR and experimental groups were determined based on GTTs (**Figure 3B**). Mice were then simultaneously switched from an HFD (60% calories from fat) to a NCD (16% calories from fat) and given an injection of 2 mg/kg of anti-BAFF Ab or an isotype control Ab every 2 weeks for a total of 6 weeks. Mice treated with the anti-BAFF Ab lost weight at the same rate as the control antibody-treated mice (**Figure 3C**). At week 14, overall glucose tolerance was improved, but no significant differences were found between the experimental groups (**Figure 3D**). However, at week 15, an ITT suggested that anti-BAFF Ab-treated mice had increased IR compared to control Ab-treated mice (**Figure 3E**). At week 17, no difference in GTT (**Figure 3F**), and at the end of the experiment, which was week 18, no difference in fed plasma insulin levels between the two groups were found (**Figure 3G**). These results suggest the anti-BAFF Ab-treated mice require more insulin to maintain glucose homeostasis, and hence, are more insulin resistant compared to the control Ab-treated mice.

The total fat mass, lean mass, gonadal WAT weight, liver weight, and total body weight of anti-BAFF Ab-treated mice trended to be higher than the control Ab-treated mice (**Figure 3H** and **Supplementary Figure 6A**). Assessment of liver steatosis revealed alanine transaminase levels were similar between the two groups (**Supplementary Figure 6B**), but liver triglyceride levels were significantly increased in the anti-BAFF Ab-treated mice (**Figure 3I**). Furthermore, there were no differences in plasma cholesterol, triglycerides, and nonesterified fatty acids (**Supplementary Figures 6C–E**). As expected, BAFF levels were significantly reduced in the plasma of anti-BAFF Ab-treated mice (**Figure 3J**).

These results suggest that BAFF neutralization during diet intervention impaired improvements in insulin sensitivity of the mice.

### 3.5 BAFF neutralization depleted B2 cells and increased inflammation in the diet intervention model

**Figure 4:**
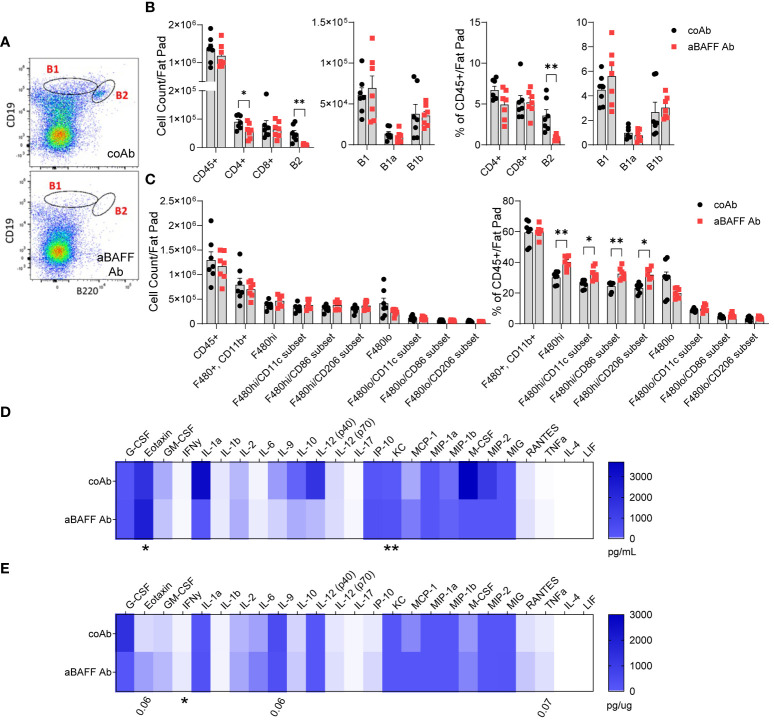
Anti-BAFF antibody treatment effectively depleted the B2 cell populations and increased inflammation in the diet intervention model. **(A)** Representative flow cytometry plots of B1 and B2 cell gating of the SVF from the gonadal WAT of control Ab and anti-BAFF Ab-treated mice. **(B)** Quantification and percent population (% of CD45+ cells) of B cell and T cell subsets in the SVF. **(C)** Quantification and percent population of macrophage subsets in the SVF of gonadal WAT. **(D)** Plasma and **(E)** gonadal WAT lysate cytokine and chemokine levels determined by the Milliplex Multiplex assay. Values are expressed as means + SEM. *, p<0.05; **, p<0.01; by parametric unpaired t-test, n=7 **(A-C)** and n=6 **(D, E)**.

To determine the effects of the anti-BAFF Ab treatment on the dynamics of adaptive and innate immune cells in various compartments, we performed flow cytometry as described for the long-term HFD model. In the spleen, blood, and peritoneal cavity of the anti-BAFF Ab-treated mice, similar to the long-term HFD diet model, B2 cell populations were effectively depleted after the transitional 1 stage, B1 cell numbers were lower, and T cell numbers were not affected (**Supplementary Figures 7A–C**). Importantly, B2 cells in the SVF of gonadal WAT were significantly depleted indicating the anti-BAFF Ab penetrated the adipose tissue and effectively neutralized BAFF (**Figures 4A, B** **).** The B1a cell number and percentage were not affected by BAFF neutralization. Interestingly, irrespective of the control Ab or anti-BAFF Ab treatment, the number and the percentage of B1b cells (to total CD45+ cells) were ~3-fold higher in the diet intervention model compared to the long-term HFD model. Since HFD does not affect B1b cell number in the frequency in the gonadal WAT (29), this result suggests active involvement of B1b B cells in WAT remodeling during diet intervention. However, anti-BAFF Ab treatment did not affect the number or the percentage of B1a and B1b cells in the SVF. CD4+ T cell numbers were partly lower, however, the percentage of CD4+ T cells and CD8+ T cells to total leukocytes was similar in the anti-BAFF Ab-treated group (**Figure 4B**) suggesting BAFF neutralization did not affect the dynamics of T cell populations in this study.

Analysis of macrophage subsets revealed no differences in the total cell counts (**Figure 4C**), however, within the CD45+ population the percent of F4/80hi macrophages, considered to be infiltrating macrophages (33), were increased and the percent of F4/80lo macrophages, considered to be resident macrophages (33), was decreased. Of the F4/80hi macrophage subset, the percent of macrophages expressing CD11c and CD86, markers of M1 macrophages, and CD206, a marker of M2 macrophage, were increased in the BAFF neutralized mice. Although the frequency of NK cells and eosinophils were similar, neutrophils were higher in the SVF of BAFF-neutralized mice (**Supplementary Figure 7D**). Analysis of inflammatory cytokines using a MILLIPLEX Multiplex Assays constating of 25 markers revealed significantly higher levels of eotaxin/C-C motif chemokine 11 (CCL11) and keratinocyte chemoattractant (KC)/chemokine (C-X-C motif) ligand 1 (CXCL1) in the plasma of anti-BAFF Ab-treated mice (**Figure 4D**). Further, the gonadal WAT lysate had increased Interferon γ (IFN-γ), and a trend towards increased eotaxin, Interleukin 9 (IL-9), and tumor necrosis factor α (TNF-α) levels in the anti-BAFF Ab-treated mice (**Figure 4E**). In fact, eotaxin and KC are potent chemoattractant for neutrophils, which may have led to increased neutrophil frequency in the gonadal WAT.

Altogether, BAFF neutralization in the diet-intervention model depleted mature B2 cells, however, increased the levels of circulating chemoattractant, increased inflammation, and the proportion of infiltrating macrophages in the gonadal WAT.

### 3.6 BAFF neutralization increased CLS formation and depleted autoantibodies that promote clearance of dead adipocytes

**Figure 5:**
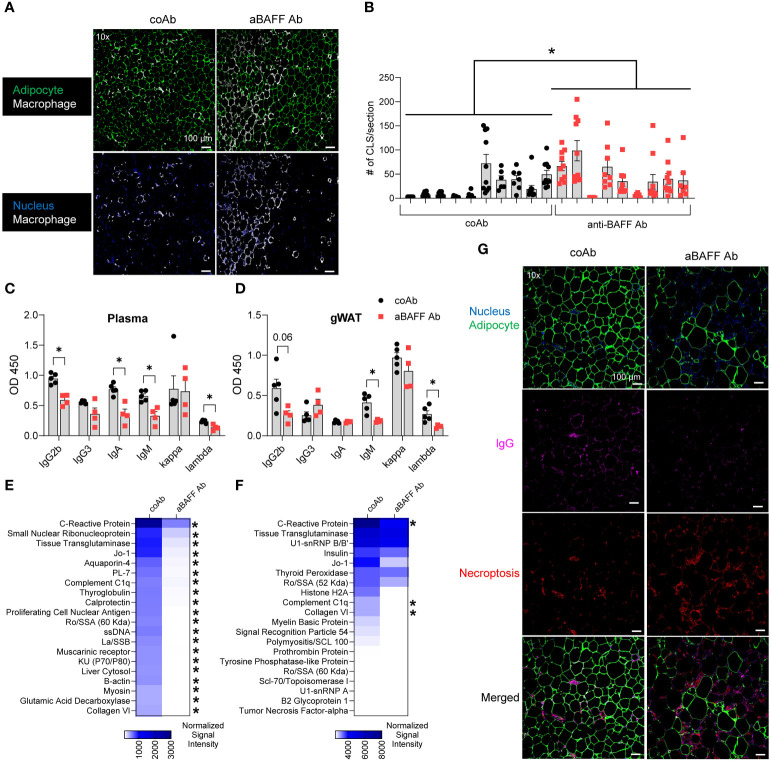
BAFF neutralization increased CLS formation and decreased autoantibody levels. **(A)** Representative confocal images of gonadal WAT from control and anti-BAFF Ab-treated groups stained for nucleus (DAPI-blue), macrophages (Mac2-white), and adipocytes (FABP4-green). **(B)** Quantification of the number of CLSs per section per mouse. **(C)** Plasma and **(D)** gonadal WAT antibody isotype levels from control and anti-BAFF Ab-treated mice. **(E)** Plasma IgG and **(F)** IgM autoAb levels in control and anti-BAFF Ab-treated mice after diet intervention. The top 20 autoAbs out of 120 tested autoAbs are shown. **(G)** Representative confocal images of gonadal WAT from control and anti-BAFF Ab-treated groups stained for nucleus (DAPI-blue), adipocytes (FABP4-green), necroptosis (phosphorylated MLKL - red), and IgG (white). Values are expressed as means + SEM. *, p<0.05; **, p<0.01; and ***, p<0.001 by a generalized estimating equation **(B)** or nonparametric U-test. n=9-10 mice, 1 section per mouse, 8-10 images per section **(A, B)**, n=4-5 mice **(C-F)**, n=4-6 mice, 1 section per mouse, 5 images per section **(G)**. Scale bars: 100 μm **(A, G)**.

The increased number of dead adipocytes, which is identified by the increased number of CLSs, is an indicator of impaired phagocytic removal of dead adipocytes (32). We, therefore, determined CLS (Mac2 positive macrophages surrounding FABP4 negative adipocytes) numbers with confocal microscopy and identified increased CLS numbers in the gonadal WAT of anti-BAFF Ab-treated mice (**Figures 5A, B**). Adipocyte size was not different between the two groups suggesting there was not an impairment in adipogenesis after BAFF neutralization (**Supplementary Figure 8A**).

Adipocytes produce BAFF and express BAFF receptors (34), and BAFF produced from adipocytes may help in the survival of adipocytes in an autocrine manner. Further, before secretion, BAFF is membrane-bound, and anti-BAFF Ab may affect adipocyte survival by binding to the membrane-bound BAFF or by neutralizing secreted BAFF. Therefore, we incubated 3T3-L1 adipocytes, which have been shown to produce BAFF (35), with the control Ab or anti-BAFF Ab and performed a mitochondrial stress test using a Seahorse extracellular flux analyzer. No differences were observed in mitochondrial respiration and aerobic glycolysis between the two groups, suggesting that the anti-BAFF Ab does not affect cellular metabolism and the survival of adipocytes (**Supplementary Figure 8B**).

Next, we examined if BAFF neutralization depleted antibodies that coat and remove the dead adipocytes by macrophages. Analysis of plasma revealed IgG2b, IgA, and IgM levels were lower in the anti-BAFF Ab treated mice (**Figure 5C**), particularly, lambda chain usage was significantly lower. The levels IgG2b, IgM, and lambda chain were also lower in the gonadal WAT of the anti-BAFF Ab-treated mice (**Figure 5D**). While the decrease in IgG2b was because of depletion of B2 cells, a decrease in IgM could be the result of decreased B1 cells in the spleen as well as depletion of the marginal zone B cells, a B2 cell subtype (**Supplementary Figure 7A**).

Autoantibody profiling of the plasma revealed that anti-BAFF Ab treatment significantly reduced the levels of multiple IgG autoAbs to antigens such as C-reactive protein, Tissue Transglutaminase, and Small Nuclear Ribonucleoprotein in the anti-BAFF Ab treated mice (**Figure 5E**). In this line, some of IgM autoAbs to antigens such as C-reactive protein, Complement C1q, and Collagen VI were depleted (**Figure 5F**). Since some of the depleted IgMs do not belong to the group of natural IgM antibodies (36), they are likely adaptive IgMs generated from splenic marginal zone and follicular B cells (37). Complement protein C1q is critical for IgM-medicated dead cell clearance (36), and interestingly, IgM antibodies against C1q were identified in the control mice, which were depleted in the anti-BAFF Ab-treated mice. We further identified more necroptotic adipocytes and less IgG deposition in the CLS of gonadal WAT of anti-BAFF Ab-treated mice compared to the control Ab-treated mice (**Figure 5G**).

**Figure 6:**
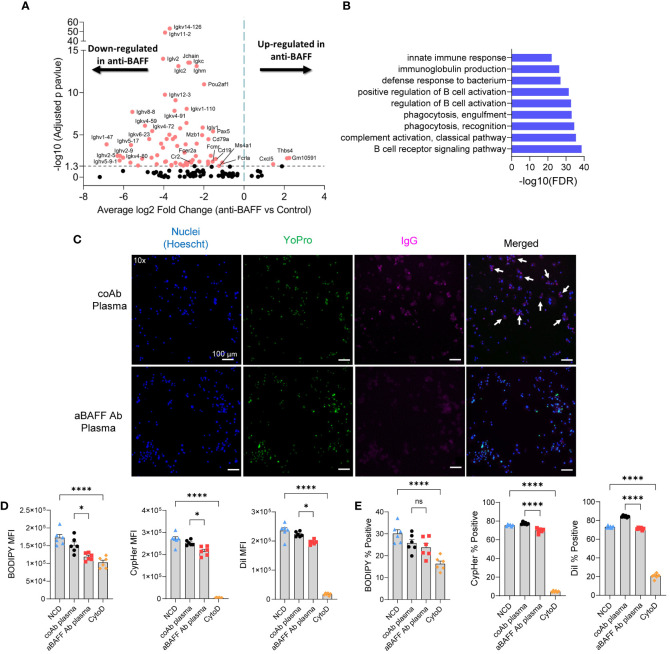
BAFF neutralization impaired phagocytosis pathways in the gonadal WAT and *in vitro*, plasma from BAFF-neutralized mice showed lesser phagocytotic activity compared to the plasma from the control mice. **(A)** Volcano plot of RNA-seq data for the SVF of diet intervention mice. Significantly up-and down-regulated genes are reported as red dots. The *P*-value threshold (0.05) is shown as a dashed line. **(B)** Gene ontology (GO) functional enrichment analysis of significant down-regulated DEGs. Top 9 GO terms of biological processes shown. The horizontal axis represents negative Log10 of the false discovery rate. **(C)** Representative fluorescent images of apoptotic adipocytes incubated with plasma from NCD or HFD-fed mice stained for nucleus (Hoechst-blue), apoptosis (YoPro-green), and IgG (pink, anti-IgG antibody). White arrows indicate cells positive for Hoechst, YoPro, and IgG staining. **(D, E)** Flow cytometry of macrophages after co-cultured with apoptotic adipocytes in the presence of IgG-rich plasma from control or anti-BAFF Ab-treated diet intervention mice. **(D)** The amount of apoptotic cell uptake was assessed by median fluorescent intensity (MFI) of BODIPY, CypHer5e, and DiI of macrophages. **(E)** The percentage of phagocytic macrophages was determined by the percent of BODIPY, CypHer5e, and DiI-positive macrophages. Values are expressed as means + SEM. *, p<0.05; ****, p<0.0001 by parametric unpaired t-test. n=4-5 (A & B), n=3 wells per treatment, 3 images each **(C)**, and n=6 **(D–E)**. Scale bars: 100 μm **(D)**.

To better understand the effect of BAFF neutralization in the cellular activities of the SVF of the gonadal WAT we utilized bulk RNA sequencing. Most down-regulated genes in the SVF of the anti-BAFF Ab-treated group were involved with B cell activation and immunoglobulin production (**Figure 6A**). Interestingly, decreased expression of multiple genes involved in the synthesis of immunoglobulin kappa light chain variable (IGKV) and immunoglobulin heavy-chain variable (IGHV) suggested a decreased repertoire of B cells after BAFF neutralization (**Supplementary Table 2**). Furthermore, gene ontology analysis of down-regulated genes revealed impairment in pathways involved with immunoglobulin production, B cell activation, and complement activation (**Figure 6B**). Importantly, pathways involved in phagocytosis recognition and engulfment were downregulated in the BAFF-neutralized mice (**Figure 6B**).

Next, as we have established that IgG autoAbs can play a role in phagocytosis we sought to determine if autoAbs for apoptotic adipocytes were present in the plasma of the mice from the diet intervention model. As expected, plasma from the control Ab-treated mice had IgG autoAbs that are specific to apoptotic adipocytes (**Figure 6C**), but not to live adipocytes (**Supplementary Figure 9A**). The anti-BAFF Ab-treated mice had decreased levels of IgG autoAbs specific to apoptotic adipocytes (**Figure 6C**). To determine if the depletion of IgG autoAbs affects phagocytosis, we utilized our *in vitro* phagocytosis assay. The IgG-rich plasma from anti-BAFF Ab-treated mice significantly decreased phagocytic uptake of the apoptotic adipocytes as demonstrated by decreased MFI (median fluorescent intensity in the macrophage gated population) of BODIPY, CypHer5e, and DiI (**Figure 6D** and **Supplementary Figure 9B**). In line with those results, there was also a decrease in the percentage of phagocytic macrophages after treatment with IgG-rich plasma from BAFF-neutralized mice (**Figure 6E**). The IgG-rich plasma from BAFF-neutralized mice did not significantly decrease the number of macrophages that took up BODIPY, though there was a downward trend (**Figure 6E**). Collectively these findings suggest that reduction of IgG autoAbs due to BAFF neutralization can impair phagocytic clearance of dead adipocytes.

## 4 Discussion

BAFF, a TNF family member and an adipocytokine, is required for the survival of B2 cells after antigen exposure. BAFF also promotes the expansion of B1b cells, but not the B1a cells (38). Elevated BAFF levels have been associated with the production of autoAbs in autoimmune diseases such as SLE, leading to the development of anti-BAFF biologics such as Belimumab which works by depleting the IgG autoAbs (39). Elevated BAFF does not affect the central tolerance of B2 cells in the bone marrow, however, allows autoreactive B2 cells to mature beyond the transitional 2 stage (T2) in the spleen (40). BAFF levels are elevated in obese individuals (41). In murine models, HFD feeding increases plasma BAFF levels and gonadal WAT is the primary producer of BAFF (42). B cells secreting IgG autoAbs specific to intracellular proteins of adipocytes have been identified in the WAT of obese and IR humans (2, 22). Here, we show that obese C57BL/6J mice, but not the lean mice produce IgG autoAbs that bind to apoptotic adipocytes. While natural IgM antibodies are known to bind to apoptotic cells and promote their clearance using C1q complement and complement receptor on the phagocyte (36), IgG autoAbs can enhance the phagocytic effect of macrophages through ADCP (28, 43). Apart from this, adaptive IgMs formed by the BAFF-dependent splenic marginal zone and follicular B cells can also promote the clearance of dead adipocytes. Our *in vitro* ADCP assay suggests that IgG-rich plasma fraction from obese mice enhanced the engulfment activity of macrophages compared to the IgG fraction from lean. This result is in line with other reports that have shown that autoAbs can promote the uptake of opsonized beads and apoptotic cells (28, 43) and promote nerve healing (21). Here, we examined if anti-BAFF Ab-mediated depletion of autoAbs affected the removal of dead adipocytes and promoted WAT inflammation using a long-term HFD feeding and a diet intervention murine model.

The etiology of diabetes in humans suggests a multistage model that is primarily based on changes in beta-cell mass and circulating insulin levels (44). Unexplained weight loss is also a common phenomenon in obese humans before they are diagnosed with diabetes (45). These findings suggest that various compensatory mechanisms are triggered to maintain glucose homeostasis in the body before the commencement of diabetes. While murine models of diet-induced obesity and diabetes do not recapitulate all compensatory mechanisms of humans, long-term HFD feeding to C57BL/6J capitalizes on a compensatory effect detailed by Strissel et al. (6) C57BL/6J mice fed an HFD for 18-20 weeks experience a transient healthy remodeling process within the adipose tissue characterized by a decrease in the number of CLSs and a partial increase in insulin sensitivity. The increases in insulin sensitivity are attributed to the clearance of dead adipocytes by macrophages and the formation of new insulin-sensitive adipocytes. In our study, following 20 weeks of HFD, we were able to recapitulate the partial gain in insulin-sensitizing effect in the control Ab-treated mice. However, this effect was attenuated in the BAFF-neutralized mice suggesting BAFF depletion attenuated healthy WAT remodeling. Furthermore, aberrant lipid handling by gonadal WAT can cause excess fat deposition in the liver leading to hepatic steatosis, another factor that contributes to systemic IR. In fact, triglyceride levels were higher in the livers of the BAFF-neutralized mice.

Similar to the long-term HFD model, the diet-intervention model also showed increased IR and increased triglyceride accumulation in the livers of the BAFF neutralized mice. Previous reports indicate that diet intervention leads to an initial increase in macrophage content in the WAT and extensive lipolysis in C57BL/6J mice (9). Detailed single-cell RNA sequencing of gonadal WAT of C57BL/6J mice after a calorie restriction, that is, lowering the amount of HFD consumption by 70%, led to the accumulation of a macrophage subpopulation which showed enhanced characteristics of phagocytosis (10). This suggests that enhanced phagocytotic activity of macrophage promtes healthy remodeling of the WAT and lower inflammation. In the diet-intervention model, BAFF neutralization increased the proportion of F4/80hi macrophages, and F4/80hi expressing M1 macrophage markers CD11c and CD86, and M2 markers CD206. No significant differences were found in the proportion of F4/80lo macrophages. F4/80hi macrophage population dominates in the gonadal WAT after 16 weeks of HFD (33), and compared to F4/80lo, F4/80hi WAT macrophages are considered to be proinflammatory and critical drivers of IR (46). Taking these together, BAFF neutralization increased the proportion of resident anti-inflammatory macrophages and recruited proinflammatory macrophages. However, this altogether increased the levels of proinflammatory makers IFN-γ and TNF-α in the gonadal WAT. Further, the proportion of neutrophils and the levels of their cogent chemoattractant eotaxin (47) were increased favoring overall skewing toward a proinflammatory microenvironment in the gonadal WAT of BAFF-neutralized mice. Bulk RNA sequencing of SVF from gonadal WAT shows upregulation of the chemokine CXCL5 which is involved in neutrophil chemotaxis. Infiltrating neutrophils forming neutrophil extracellular traps (NETs) are deleterious in many diseases such as SLE (48) and Type 1 diabetes mellitus (49). More studies are needed to determine if increased neutrophil infiltration during IR may result in NET formation causing adipocyte death. Apart from this, natural IgMs to oxidation-specific epitopes can lower inflammation during HFD-induced obesity (31). Further, lower IgM levels in the BAFF-neutralized mice may contribute to increased inflammation in the gonadal WAT.

**Figure 7:**
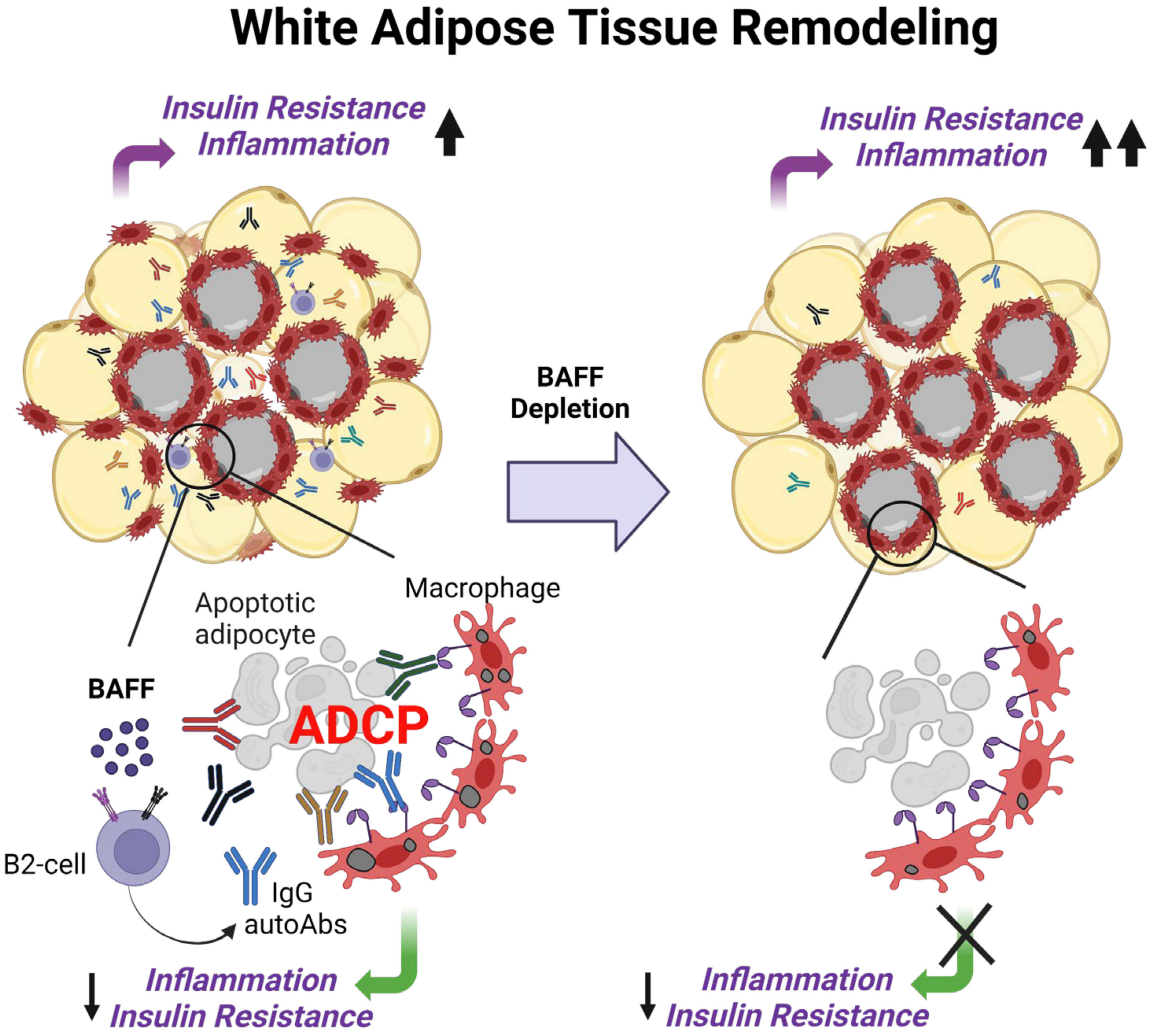
Working model of BAFF’s role in the regulation of insulin resistance during healthy remolding of white adipose tissue. Excess BAFF promotes the survival of autoreactive B2 cells leading to the production of IgG autoantibodies against dead adipocytes. During healthy remodeling of the white adipose tissue, these IgG autoantibodies, natural and adaptive IgM antibodies bind to the dead adipocytes, promote their phagocytic clearance by macrophages, and lower inflammation and IR. The introduction of a BAFF-neutralizing antibody causes depletion of adipocyte-binding autoantibodies, dysregulating remodeling the white adipose tissue, and impairing the recovery from insulin resistance.

Consistent with delayed IR recovery in the diet intervention BAFF-neutralized mice, we observed increased dead adipocytes in the gonadal WAT. However, IgG deposition was reduced in the adipose tissue and IgG autoAbs were depleted in the plasma of these mice. Some of the highly synthesized IgG autoAbs that were depleted in the BAFF-neutralized mice target C-reactive protein, tissue transglutaminase, aquaporin 4, and complement C1q, and these proteins are associated with obesity-induced WAT inflammation and increase in IR (50–53). Interestingly, autoAbs to small nuclear ribonucleoprotein, Sjögren’s-syndrome-related antigen A (Ro/SSA), Jo-1, and synthetase syndrome (PL)-7 which are normally detected in SLE and other autoimmune diseases (54), were also identified in high levels in the plasma of the control mice and were depleted after BAFF neutralization. Our *in vitro* ADCP experiment revealed that macrophages, in the presence of an IgG-rich plasma fraction from BAFF-neutralized mice had decreased phagocytosis compared to control mice. The bulk RNA sequencing of the SVF of WAT revealed a decreased repertoire of B cells which may reduce generation of autoAbs. Further phagocytosis pathways were attenuated supporting our hypothesis that autoAbs promote phagocytosis during IR recovery. Our study fits into a model in which BAFF neutralization leads to the depletion of autoAbs that promotes phagocytic clearance of dead adipocytes in the WAT, impairs the resolution of inflammation, and increases systemic IR (**Figure 7**). The excess lipid spillover from WAT can be taken up by the liver causing triglyceride accumulation in the BAFF-neutralized mice.

According to previous reports genetic deficiency of *Baff* protects against the development of HFD-induced IR irrespective of weight gain (12, 55). *Baff-*deficient mice exhibit a “healthy obesity” phenotype, in which their total body weight is similar to wild-type obese mice, however, they are protected from IR. This healthy obesity effect is due to the redistribution of adipose tissue mass away from the gonadal WAT to the subcutaneous WAT (55, 56). In our study, we did not observe any change in adiposity. This may be because the genetically *Baff*-deficient mice are not exposed to BAFF in their lifetime and feeding HFD activates multiple compensatory mechanisms preventing IR, such as adipose tissue redistribution. In agreement with *Baff-*deficient mice, a study by Shen et al. showed that BAFF neutralization with an anti-BAFF Ab partly reduces IR in diet-induced obese C57BL/6J mice (29). In this study, the anti-BAFF Ab was administered 6 weeks after HFD in contrast to 12 weeks after HFD in our study. Furthermore, the anti-BAFF Ab used was a hamster anti-mouse BAFF Ab (10F4, GlaxoSmithKline), whereas we used the Sandy-2 clone (Adipogen). WAT dysfunction, such as adipocyte hypertrophy and death is significantly lower after 6 weeks on HFD compared to 12 weeks on HFD (6). BAFF neutralization sufficiently decreased the IgGs (29). Since the Fc portion of the IgGs can bind to Fc receptors on macrophages and promote inflammation *via* TNF-α secretion (2), a decrease in the levels of circulating IgGs in the 6-week HFD model may decrease WAT inflammation and IR. The 6-week HFD model, and our long-term and the diet-intervention models, together highlight that BAFF neutralization in early and in the late stages of obesity have opposing results. In support of the harmful effects of BAFF depletion, Tsiantoulas et al. have reported that BAFF neutralization was not supportive of high-fat atherogenic diet-induced atherosclerosis in mice (57). Further, Saidoune et al. have reported that depending on the circulating cholesterol levels, BAFF neutralization has opposing effects despite protection from lupus in mice (58).

Apart from B cells, BAFF receptors are expressed in many cell types, such as T cells and adipocytes (59, 60). Although more studies are needed to determine the role of T cell activation in the BAFF-neutralized mice, our results show no significant differences in the CD4+ T cell and CD8+ T cell frequencies in the gonadal WAT. Furthermore, BAFF has been shown to affect lipid handling in primary adipocytes from subcutaneous WAT, and further studies are warranted to better understand the effect of BAFF neutralization on adipocytes isolated from gonadal WAT (60). The impact of aging, which has a well-established effect on the enhancement of dysmetabolism, will be an important factor to study in future studies. A recent report by Yu et al. showed that in aged (12-month-old) C57BL/6J mice, IgG levels are increased which can activate macrophages to induce fibrosis in gonadal WAT and increase systemic IR (61). It remains to be determined if aging promotes the generation of autoreactive B2 cells and IgG autoantibodies, and if BAFF depletion plays a role.

The long-term HFD feeding model has some limitations. First, BAFF neutralization depleted B2 cell subtypes in multiple compartments, however, in contrast to the diet-intervention mice, B2 cell subtypes in the gonadal WAT were unaffected in the long-term HFD model. The gonadal WATs of the long-term HFD-fed mice weigh ~2000 mg (**Supplementary Figure 2A**), while the gonadal WAT from the diet-intervention mice weigh approximately ~1000 mg (**Supplementary Figure 6A**). HFD-induced obesity significantly decreases vascularization (62) and promotes fibrosis (63) of gonadal WAT, which can affect the penetrance of the anti-BAFF Ab into the gonadal WAT. Weight loss programs, such as exercise, attenuate WAT fibrosis (63). In our diet-intervention model, the mice significantly lost weight, and were likely to have increased vascularization and decreased fibrosis facilitating penetrance of the anti-BAFF Ab. Furthermore, the larger size of the gonadal WATs in the long-term HFD mice can produce more BAFF than the smaller WATs of the diet-intervention mice. This suggests more sequestering of the anti-BAFF Ab to large adipocytes in the gonadal WAT of long-term HFD mice and less availability of the antibody to gonadal WAT resident B cells. This leads to less depletion of B cells in the gonadal WAT of long-term HFD mice despite that the quantity of the anti-BAFF Ab injected was normalized to the body weight of the mice.

Second, apart from lack of depletion of B2 cells in the WAT, BAFF neutralization did not affect the immunoglobulin levels in the plasma of long-term HFD mice. Whereas BAFF neutralization significantly decreased IgG2b, IgA, and IgM in the plasma of the diet-intervention mice. In the long-term HFD model, we did not assess the B cell populations within the inguinal lymph nodes (gonadal WAT draining lymph nodes) and bone marrows which can contribute to the plasma immunoglobulin levels. Moreover, since B cells were not depleted in the gonadal WAT, we speculate that B cells were also retained in the fat-associated lymphoid clusters in the omental fat (64). Frasca et al. reported that B cells within the adipose tissue produce antibodies (22). Furthermore, in the long-term HFD-fed mice, overnutrition can cause the survival of unknown antibody-secreting B cell populations (65), which were not responsive to anti-BAFF Ab treatment. Nonetheless, anti-BAFF Ab treatment depleted IgG and IgM autoantibodies in the plasma (**Supplementary Figures 4G, H**).

Third, lack of differences in the CLS number in the gonadal WAT of control Ab *vs* the anti-BAFF Ab-treated mice. As reported previously (6), we observed that the insulin sensitivity of the control Ab-treated mice was significantly improved from week 16 to week 20. While we did not collect tissue at week 16 and week 20, this increase in insulin sensitivity is attributed to extensive WAT remodeling, that is, removal of dead adipocytes, and BAFF neutralization impaired this process. However, this healthy WAT remodeling and increase in insulin sensitivity is a transient process as the mice keep receiving HFD. In fact, mice on an HFD for 24 weeks have significantly increased IR (66, 67). In our study, as the mice were euthanized at 23 weeks on HFD, the critical difference between CLS counts in the gonadal WAT is lost. However, as excess lipid spillover from WAT deposits in other organs, such as the liver (68), we found increased triglyceride accumulation in the livers of BAFF-neutralized mice.

In the diet intervention model, healthy remodeling of WAT of obese mice was induced by switching HFD to NCD, thus lowering the calorie intake. BAFF neutralization resulted in a reduced ability to recover from IR based on the ITT at 3 weeks after the diet intervention. In contrast, the GTT at 5 weeks after the diet switch did not show significant differences between the two groups, plasma insulin levels in the fed state were also not significantly different between the two groups. In the case of the ITT, the amount of insulin injected into the mouse is controlled, allowing us to assess insulin utilization based on glucose uptake. In the GTT, insulin production is not controlled, therefore the BAFF-neutralized mice compensated for the decrease in insulin sensitivity by producing more insulin in response to the glucose injection compared to the control mice (69). Furthermore, gut microbiota affects obesity and IR, and diet intervention affects gut microbiota (70). It is unknown how BAFF neutralization affects gut microbiota. Altogether the effects of BAFF neutralization and diet intervention may account for some of the discrepancies noted between the two models in regard to insulin production.

This study provides the first evidence that IgG autoAbs generated during high-fat diet-induced obesity bind to dead adipocytes and promote their phagocytic clearance. This serves as a compensatory repair mechanism for excessive cell death in the white adipose tissue. Removal of these autoAbs by BAFF neutralization impairs healthy remodeling of the white adipose tissue and exacerbates insulin resistance in mice. As murine models do not fully recapitulate human physiology, studies on Belimumab-receiving human subjects are required to determine the role of BAFF depletion on tissue remodeling and insulin resistance. This study highlights the need for a better understanding of the role of autoAbs and how BAFF biologics affect tissue remodeling processes and insulin sensitivity in patients.

## Data Availability

The raw bulk RNA-sequencing data reported in this paper has been deposited in the National Center for Biotechnology Information (NCBI) database and is publicly accessible at GSE255950.
