# Supplementary material for: BAFF neutralization impairs the autoantibody-mediated clearance of dead adipocytes and aggravates obesity-induced insulin resistance
Source: Front Immunol. 2024 Aug 9;15:1436900. doi: 10.3389/fimmu.2024.1436900 (PMC11341376; doi:10.3389/fimmu.2024.1436900)
Supplement: Supplementary file 1 [file DataSheet_1.pdf]

Supplementary Figure 1

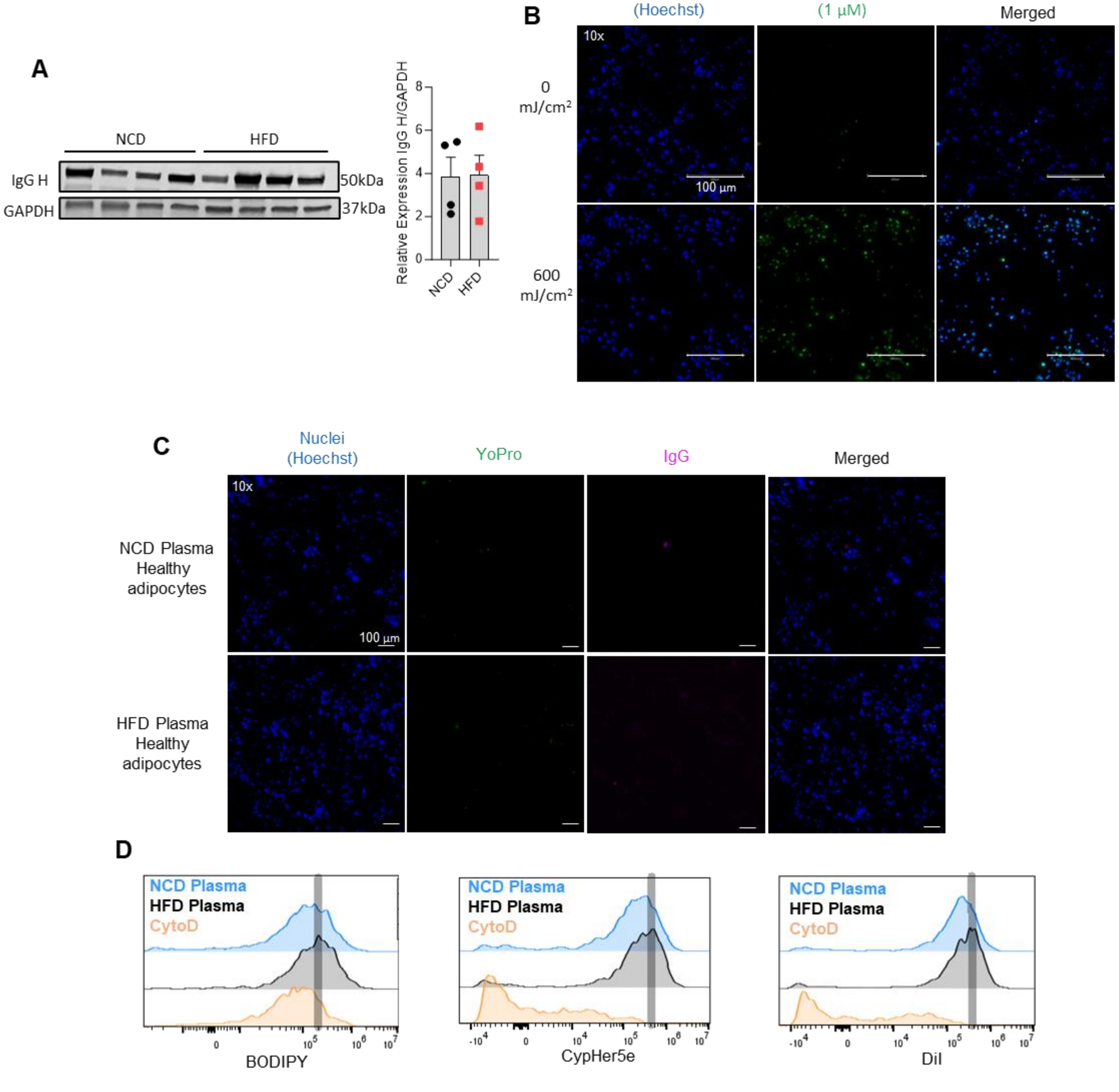


**Supplementary Figure 1 (Supplement to Figure 1). Binding of IgG autoantibodies to healthy adipocytes and representative flow cytometry plots of phagocytosis assay. (A)**Western blot and quantification of IgG and GAPDH from gWAT of WT mice on NCD or HFD from 11 weeks (**B**) Representative fluorescent images of 3T3-L1 adipocytes irradiated with 0 or 600 mJ/cm$^2$ of UV radiation and stained for nucleus (Hoechst-blue) and apoptosis (YoPro-green). **(C)** IgG autoantibodies from obese mice do not bind to healthy 3T3-L1

adipocytes. Representative fluorescent images of healthy adipocytes incubated with plasma from NCD or HFD-fed mice stained for nucleus (Hoescht-blue), apoptosis (YoPro-green), and IgG (pink). **(D)** Flow cytometry of macrophages and apoptotic adipocytes after co-cultured with IgG-rich plasma from NCD and HFD fed mice. Histograms of median fluorescent intensity (MFI) of BODIPY, CypHer5e, and DiI of macrophages. n=3 wells per treatment, 3 images each **(B and C)**, n=6 **(D)**. Scale bars: 100 μm **(B and C)**

## Supplementary Figure 2

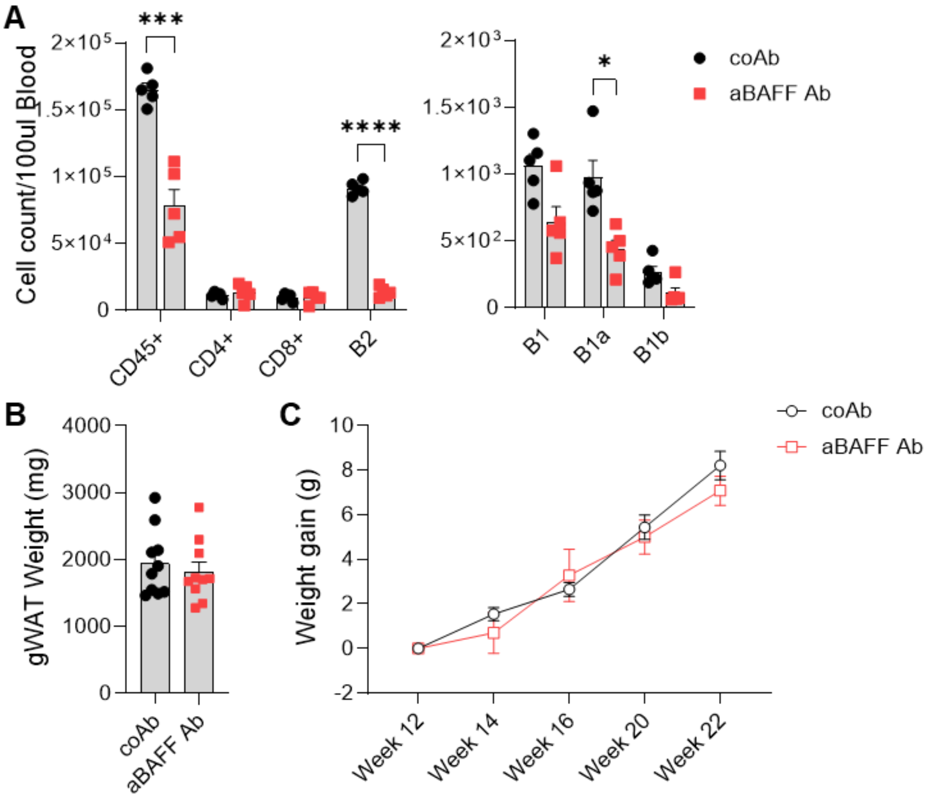


**Supplementary Figure 2. (Supplement to Figure 2 for the long-term high-fat diet model) (A)** Quantification and percent population of B cell and T cell subsets in the blood of long-term HFD mice on 16 weeks on HFD. **(B)** Weight of total gonadal white adipose tissue (WAT). **(C)** Weight gain of control and anti-BAFF antibody-treated mice during HFD. Values are expressed as means + SEM. *, $p<0.05$; ***, $p<0.001$; and ****, $p<0.0001$by parametric unpaired t-test. n=5 **(C)** and n=9-10 **(B & C)**.

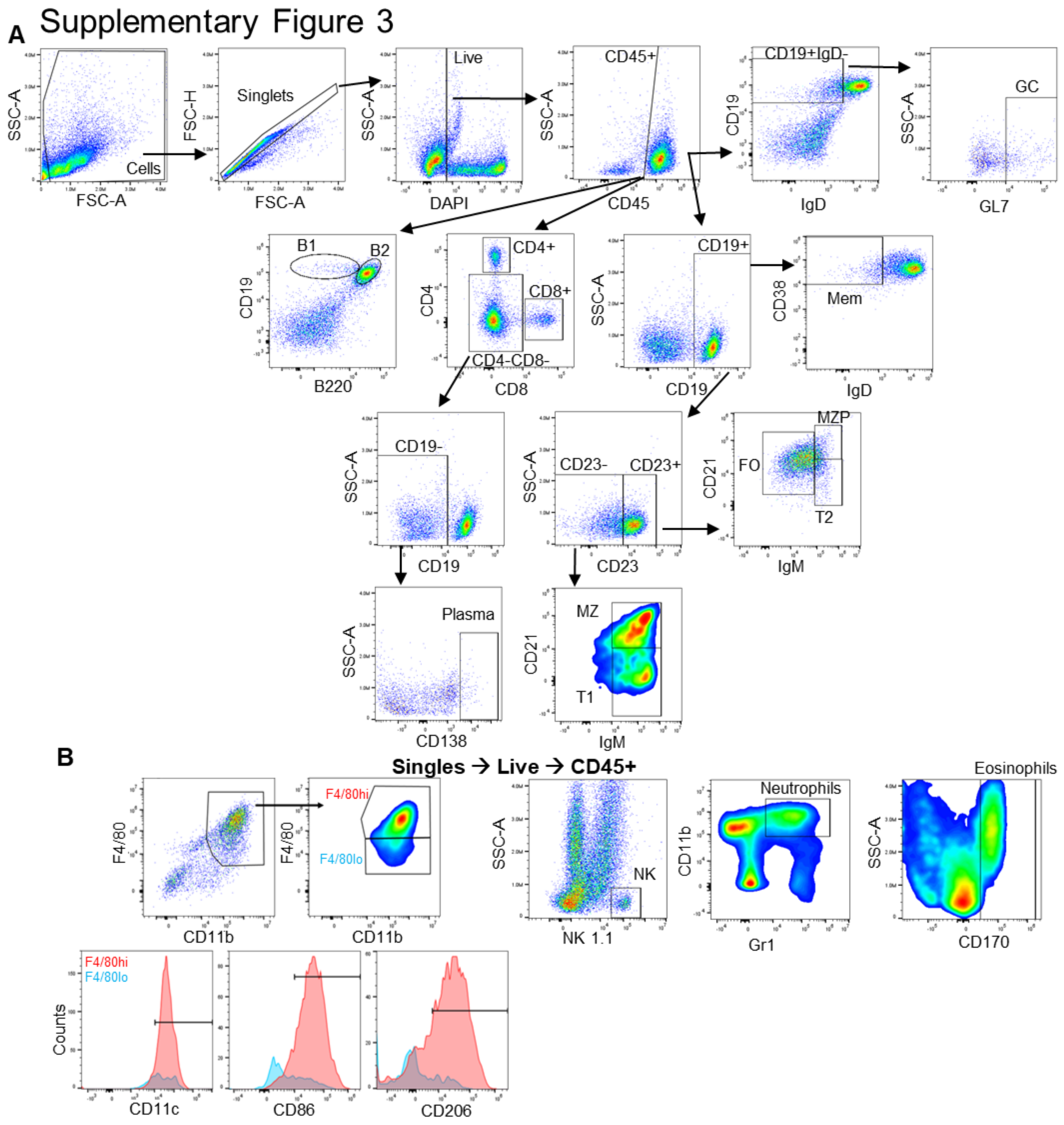


**Supplementary Figure 3. Flow Cytometry Gating Strategy. (A)** Gating strategy for phenotyping of B-cell subsets B1, B2, memory (Mem), marginal zone (MZ), transitional 1 (T1), marginal zone progenitor (MZP), follicular (FO), transitional 2 (T2), germinal center (GC), and plasma B cells was developed using splenocytes from C57BL/6J mice. **(B)** Gating strategy for phenotyping macrophage subsets, NK cells, neutrophils, and eosinophils was developed using stromal vascular fraction from gonadal white adipose tissue of C57BL/6J mice.

# Supplementary Figure 4

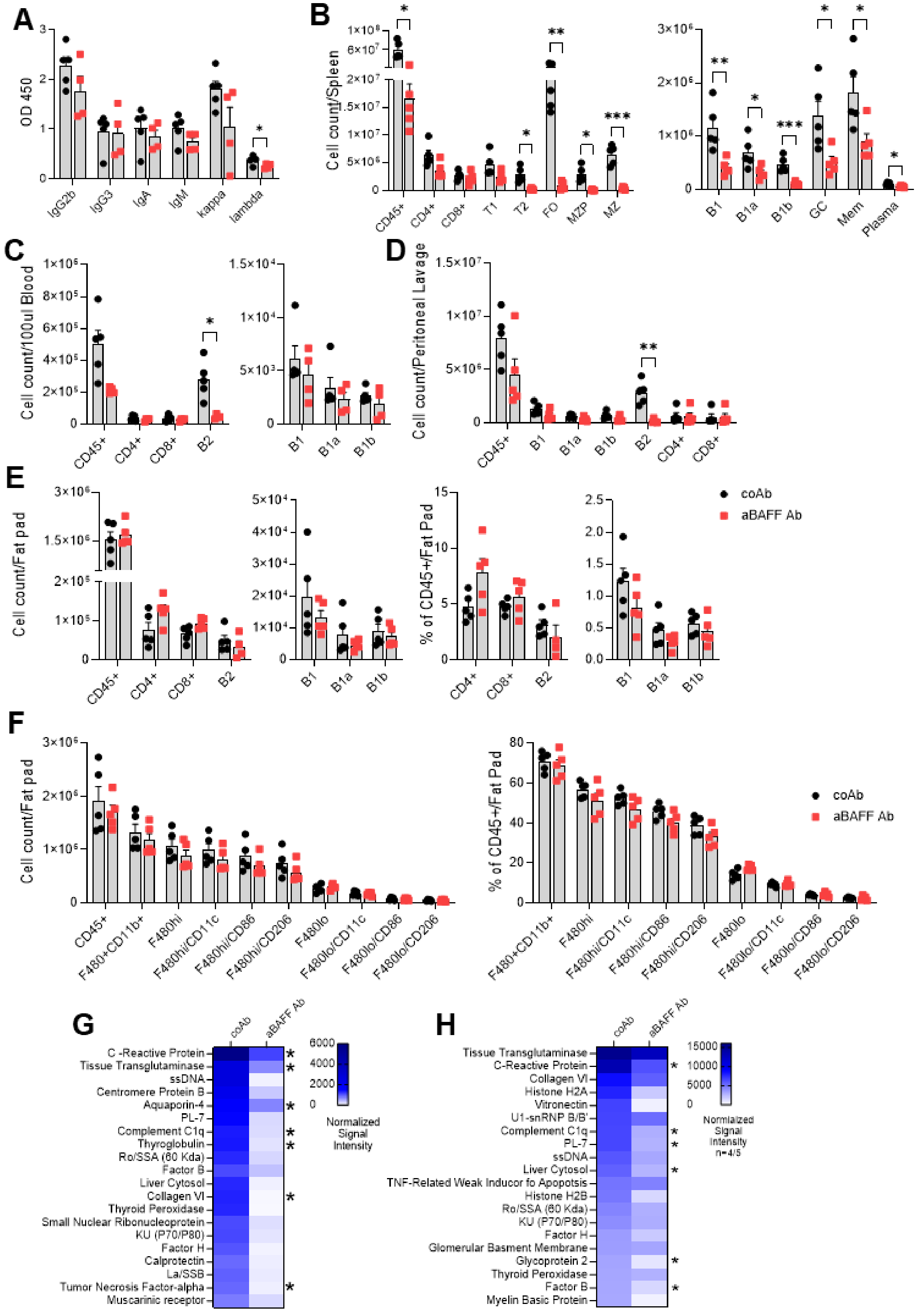

**Supplementary Figure 4. B2 B cells were retained in the gonadal WAT of anti-BAFF antibody treated mice in the long-term high-fat diet model. (A)** Levels of immunoglobulin isotypes in the plasma of the control and anti-BAFF antibody-treated mice. Quantification of B cell and T cell subsets in the spleen **(B)**, blood **(C)**, and peritoneal lavage **(D)** of the long-term HFD mice. **(E)** Quantification and percent population (% of CD45+ cells) of B cell and T cell subsets in the SVF of gonadal WAT. **(F)** Quantification and percent population of macrophage subsets in the SVF of gonadal WAT. **(G-H)** Plasma IgG **(G)** and IgM **(H)** autoantibody levels in control and anti-BAFF antibody-treated mice. Autoantibodies for top 20 out of 120 antigens are shown. Values are expressed as means + SEM. *, $p<0.05$; **, $p<0.01$; by parametric unpaired t-test or nonparametric t-test (U-test), n=4-5 **(A, G, & H)** and n=5 **(B-F)**.

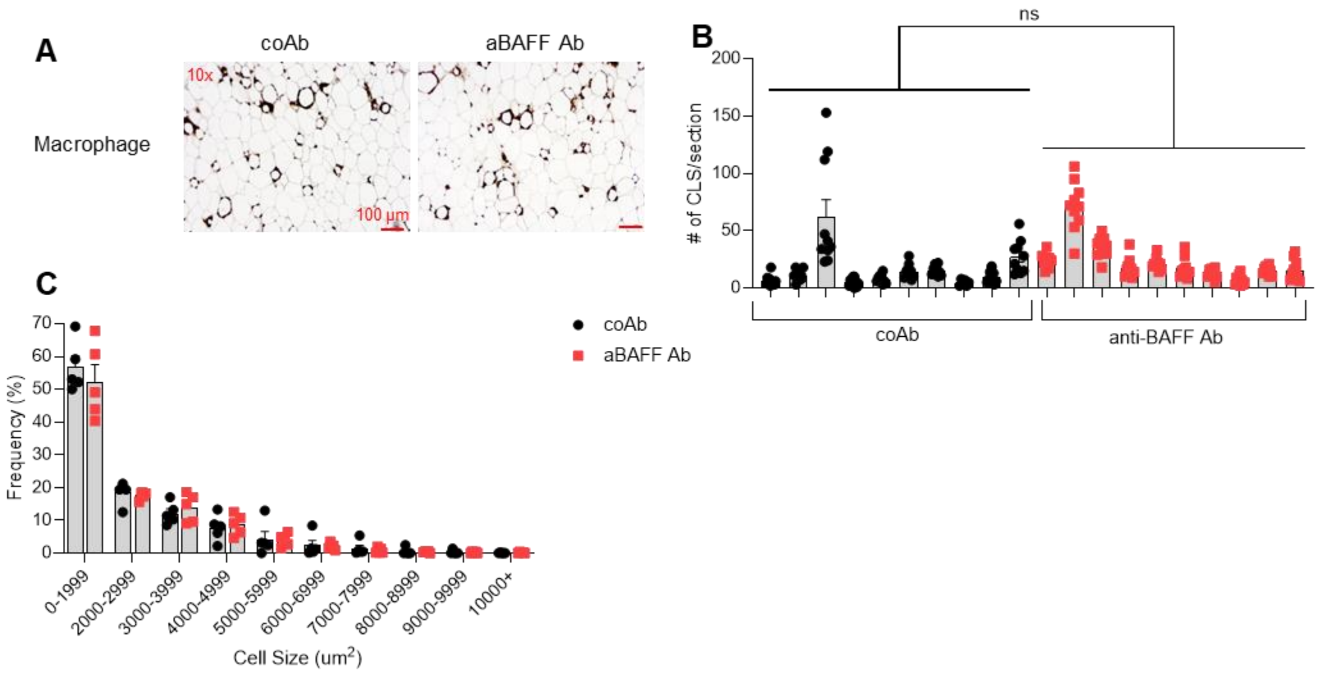


**Supplementary Figure 5. Remodeling of gonadal WAT in the long-term high-fat diet model. (A)** Representative images of gonadal WAT from control and anti-BAFF Ab-treated mice stained for macrophages (Mac2-brown). **(B)** Quantification of the number of crown-like structures per section per mouse. **(C)** Quantification of adipocyte size by ImageJ. Values are expressed as means + SEM. ns, not significant by a generalized estimating equation. n=10 mice, 1 section per mouse, 8-10 images per section **(A-B)**, n=5 mice, 1 section per mouse, 8-10 images per section **(C).** Scale bars: 100 µm **(A)**.

## Supplementary Figure 6

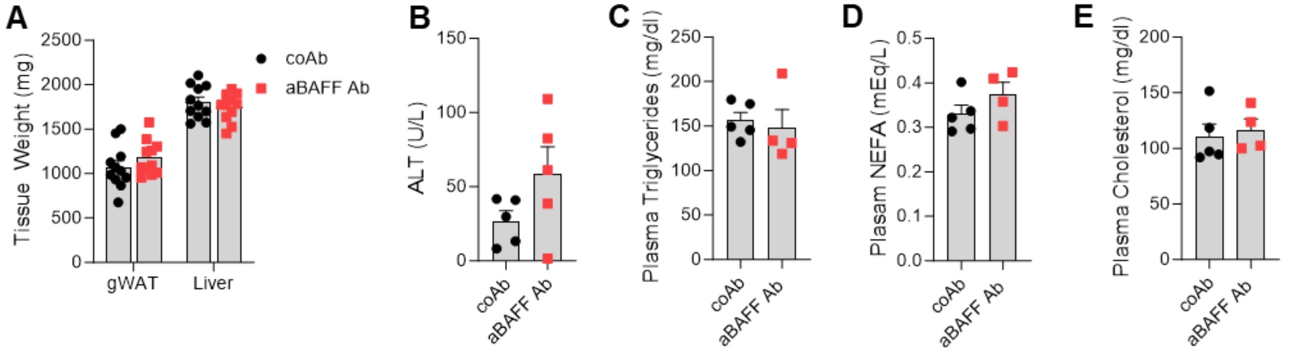


**Supplementary Figure 6. (Supplement to Figure 3 for the diet intervention model).** **(A)** Liver and gonadal WAT weights mice treated with control or anti-BAFF antibody. Plasma levels of alanine transaminase **(B)**, triglycerides **(C)**, non-esterified fatty acids **(D)**, and cholesterol **(E)**. Values are expressed as means + SEM. n=10-11 **(A)**, and n=4-5 **(B-E)**.

# Supplementary Figure 7

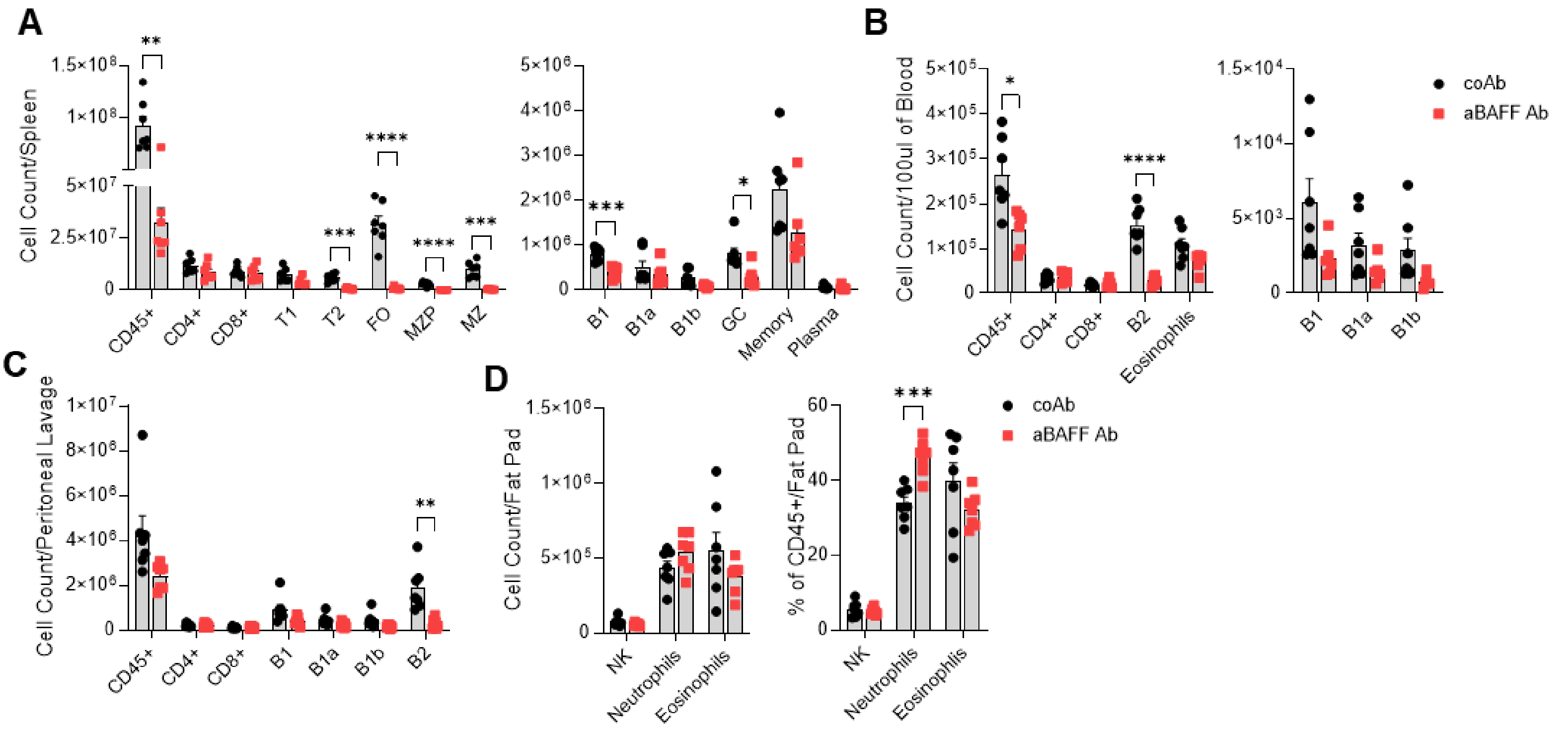


**Supplementary Figure 7. (Supplement to Figure 4 for the diet intervention model).** Quantification of B cell and T cell subsets in the spleen **(A)**, blood **(B)**, and peritoneal lavage **(C)** of the diet intervention mice. **(D)** Quantification and percent population of NK cells, neutrophils, and eosinophils in the SVF of gonadal WAT. Values are expressed as means + SEM. *, $p<0.05$; **, $p<0.01$; by parametric unpaired t-test. n=7

# Supplementary Figure 8

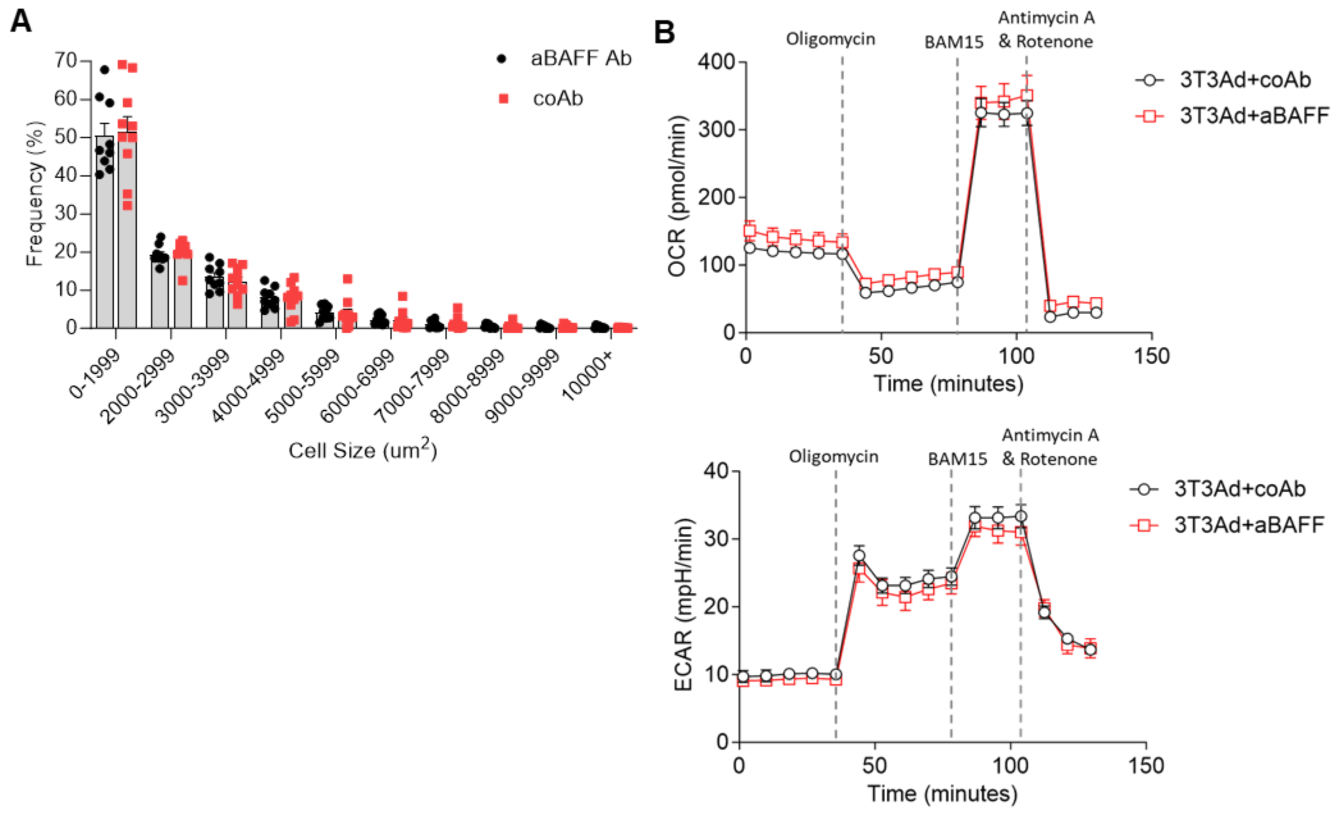


**Supplementary Figure 8. (A)** Quantification of adipocyte size by ImageJ. **(B)** Cellular respiration of adipocytes after treatment with a control antibody or anti-BAFF antibody was determined by a mitochondrial stress test on a Seahorse XF24 Analyzer. Values are expressed as means + SEM. n=9-10 mice, 1 section per mouse, 8-10 mages per section **(A),** n=5 **(B)**.

## Supplementary Figure 9

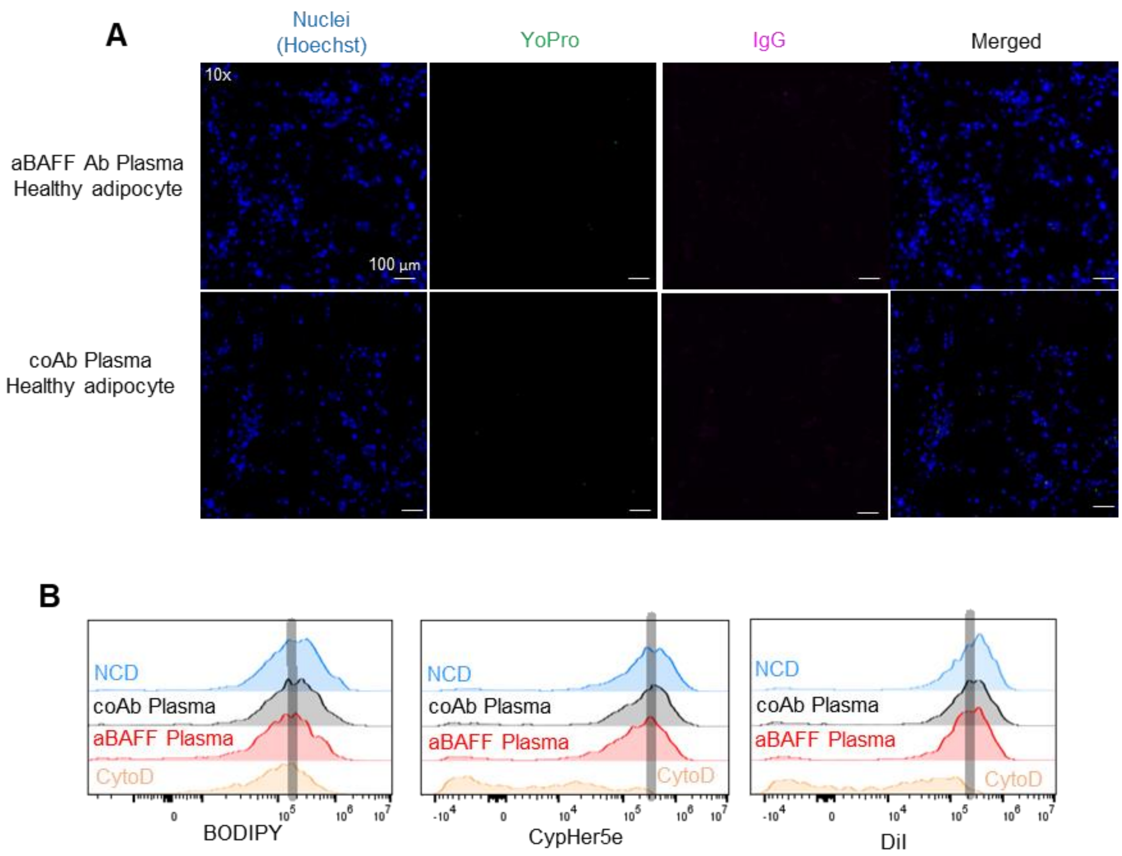


**Supplementary Figure 9. (A)** Representative fluorescent images of healthy adipocytes incubated with plasma from control antibody treated or anti-BAFF antibody treated diet intervention mice stained for nucleus (Hoescht-blue), induction of apoptosis (YoPro-green), and IgG (pink). **(B)** Flow cytometry of macrophages and apoptotic adipocytes after co-cultured with IgG-rich plasma from NCD and HFD fed mice. Histograms of median fluorescent intensity (MFI) of BODIPY CypHer5e, and DiI of macrophages. n=3 wells per treatment, 3 images each **(A)**, n=6 **(B)**. Scale bars: 100 µm **(A)**

**Supplementary Table 1. Antigens of the Autoantibody Array**

| | | | |
|---|---|---|---|
| Aggrecan | dsDNA | Ig control 2 | Proteoglycan |
| AGTR | EBNA1 | Ig control 3 | Prothrombin protein |
| Alpha Fodrin | Elastin | Ig control 4 | Rheumatoid factor (RF) |
| Alpha-actinine | Factor B | IL-12 | Ribo Phosphoprotein P0 |
| Amyloid | Factor D | IL-15 | Ribo Phosphoprotein P1 |
| anti-Ig control 1 | Factor H | Insulin | Ribo Phosphoprotein P2 |
| anti-Ig control 2 | Factor I | Jo-1 | Ro/SSA (52 Kda) |
| anti-Ig control 3 | Factor P | KU (P70/P80) | Ro/SSA (60 Kda) |
| anti-Ig control 4 | Fibrinogen IV | La/SSB | S100 |
| AQP4 | Fibrinogen S | Laminin | Scl-70/Topoisomerase I |
| B2 glycoprotein 1 | Fibronectin | LC1 | Sm |
| B2-microglobulin | GAD65 | LPS | Sm/RNP |
| B-actin | GBM | M2 | SmD |
| BPI | GDF-15 | MDA5 | SP100 |
| Calprotectin | GM-CSF | Mi-2 | Sphingomyelin |
| Cardolipin | GP2 | Mitochondrial antigen | SRP54 |
| CD4 | GP210 | MPO | ssDNA |
| CD8 | Hemocyanin | Muscarinic receptor | ssRNA |
| CENP-A | Heparan Sulphate | Myelin basic protein (MBP) | TGF-B1 |
| CENP-B | Heparin | Myosin | Thyroglobulin |
| Chondroitin Sulfate C | Histone H1 | Myosin heavy & light chain | TIF1 gama |
| COL17A1 | Histone H2A | Neuropilin-1 | TNF-a |
| Collagen I | Histone H2B | Nucleolin | TPO |
| Collagen II | Histone H3 | Nucleosome antigen | Troponin-I |
| Collagen III | Histone H4 | Nup 62 | TTG |
| Collagen IV | IA2 | PCNA | Tweek |
| Collagen V | IFN-A1 | Phosphatidyl-serine | U1-snRNP 68/70 |
| Collagen VI | IFN-B | PL-12 | U1-snRNP A |
| complement C1q | IFN-E | PL-7 | U1-snRNP B/B' |
| complement C3/C3a | IFN-L2 | PM/Scl 100 | U1-snRNP C |
| Core Histone | IFN-r | PM/Scl-75 | Vimentin |
| CRP | Ig control 1 | PR3 | Vitronectin |

## Supplementary Table 2. Differential gene expression (anti-BAFF Ab *vs* Control Ab) in the stromal vascular fraction of gonadal white adipose tissue of mice in the diet intervention model.

| log2FoldChange | padj | gene_name | gene_biotype | gene_description |
|---|---|---|---|---|
| -3.701 | 5.20E-54 | Igkv14-126 | IG_V_gene | immunoglobulin kappa variable 14-126 [Source:MGI Symbol;Acc:MGI:3643131] |
| -3.935 | 1.07E-49 | Ighv11-2 | IG_V_gene | immunoglobulin heavy variable V11-2 [Source:MGI Symbol;Acc:MGI:4947968] |
| -4.033 | 1.01E-14 | Iglv2 | IG_V_gene | immunoglobulin lambda variable 2 [Source:MGI Symbol;Acc:MGI:99548] |
| -2.773 | 2.85E-14 | Jchain | protein_coding | immunoglobulin joining chain [Source:MGI Symbol;Acc:MGI:96493] |
| -2.699 | 2.85E-14 | Igkc | IG_C_gene | immunoglobulin kappa constant [Source:MGI Symbol;Acc:MGI:96495] |
| -2.363 | 7.35E-14 | Ighm | IG_C_gene | immunoglobulin heavy constant mu [Source:MGI Symbol;Acc:MGI:96448] |
| -3.269 | 7.35E-14 | Iglc2 | IG_C_gene | immunoglobulin lambda constant 2 [Source:MGI Symbol;Acc:MGI:99547] |
| -1.991 | 1.06E-11 | Pou2af1 | protein_coding | POU domain, class 2, associating factor 1 [Source:MGI Symbol;Acc:MGI:105086] |
| -3.937 | 1.79E-10 | Ighv12-3 | IG_V_gene | immunoglobulin heavy variable V12-3 [Source:MGI Symbol;Acc:MGI:3646760] |
| -3.414 | 8.01E-10 | Igkv1-117 | IG_V_gene | immunoglobulin kappa variable 1-117 [Source:MGI Symbol;Acc:MGI:4439721] |
| -2.857 | 8.41E-09 | Igkv1-110 | IG_V_gene | immunoglobulin kappa variable 1-110 [Source:MGI Symbol;Acc:MGI:4439558] |
| -3.464 | 1.43E-08 | Igkv4-91 | IG_V_gene | immunoglobulin kappa chain variable 4-91 [Source:MGI Symbol;Acc:MGI:3642277] |
| -5.560 | 1.94E-08 | Ighv8-8 | IG_V_gene | immunoglobulin heavy variable 8-8 [Source:MGI Symbol;Acc:MGI:3815333] |
| -2.867 | 3.93E-07 | Igkv15-103 | IG_V_gene | immunoglobulin kappa chain variable 15-103 [Source:MGI Symbol;Acc:MGI:96513] |
| -4.934 | 8.23E-07 | Igkv4-59 | IG_V_gene | immunoglobulin kappa variable 4-59 [Source:MGI Symbol;Acc:MGI:3646808] |
| -2.060 | 1.39E-06 | Iglv1 | IG_V_gene | immunoglobulin lambda variable 1 [Source:MGI Symbol;Acc:MGI:96530] |
| -3.178 | 1.68E-06 | Ighv7-3 | IG_V_gene | immunoglobulin heavy variable 7-3 [Source:MGI Symbol;Acc:MGI:4439766] |
| -4.572 | 3.54E-06 | Igkv4-72 | IG_V_gene | immunoglobulin kappa chain variable 4-72 [Source:MGI Symbol;Acc:MGI:2686345] |
| -1.544 | 3.91E-06 | Pax5 | protein_coding | paired box 5 [Source:MGI Symbol;Acc:MGI:97489] |
| -3.720 | 9.70E-06 | Igkv1-135 | IG_V_gene | immunoglobulin kappa variable 1-135 [Source:MGI Symbol;Acc:MGI:3819952] |
| -2.086 | 1.09E-05 | Mzb1 | protein_coding | marginal zone B and B1 cell-specific protein 1 [Source:MGI Symbol;Acc:MGI:1917066] |
| -3.436 | 1.78E-05 | Ighv6-6 | IG_V_gene | immunoglobulin heavy variable 6-6 [Source:MGI Symbol;Acc:MGI:4439619] |
| -3.856 | 3.07E-05 | Ighv2-2 | IG_V_gene | immunoglobulin heavy variable 2-2 [Source:MGI Symbol;Acc:MGI:4439894] |
| -3.546 | 3.15E-05 | Ighv3-6 | IG_V_gene | immunoglobulin heavy variable 3-6 [Source:MGI Symbol;Acc:MGI:4439856] |
| -4.723 | 3.15E-05 | Igkv6-23 | IG_V_gene | immunoglobulin kappa variable 6-23 [Source:MGI Symbol;Acc:MGI:3711980] |
| -1.773 | 3.31E-05 | Cd79a | protein_coding | CD79A antigen (immunoglobulin-associated alpha) [Source:MGI Symbol;Acc:MGI:101774] |
| -2.356 | 1.09E-04 | Igkv19-93 | IG_V_gene | immunoglobulin kappa chain variable 19-93 [Source:MGI Symbol;Acc:MGI:107617] |
| -4.129 | 1.09E-04 | Igkv4-68 | IG_V_gene | immunoglobulin kappa variable 4-68 [Source:MGI Symbol;Acc:MGI:2686265] |
| -2.908 | 1.31E-04 | Ighv1-78 | IG_V_gene | immunoglobulin heavy variable 1-78 [Source:MGI Symbol;Acc:MGI:4439736] |
| -6.844 | 1.32E-04 | Ighv1-47 | IG_V_gene | immunoglobulin heavy variable 1-47 [Source:MGI Symbol;Acc:MGI:4439890] |
| -5.631 | 1.58E-04 | Ighv5-17 | IG_V_gene | immunoglobulin heavy variable 5-17 [Source:MGI Symbol;Acc:MGI:4439533] |
| -3.329 | 2.05E-04 | Igkv8-30 | IG_V_gene | immunoglobulin kappa chain variable 8-30 [Source:MGI Symbol;Acc:MGI:3642250] |
| -4.037 | 3.38E-04 | Ighv1-69 | IG_V_gene | immunoglobulin heavy variable 1-69 [Source:MGI Symbol;Acc:MGI:4439632] |
| -2.965 | 1.26E-03 | Fcer2a | protein_coding | Fc receptor, IgE, low affinity II, alpha polypeptide [Source:MGI Symbol;Acc:MGI:95497] |
| -4.323 | 2.58E-03 | Ighv1-72 | IG_V_gene | immunoglobulin heavy variable 1-72 [Source:MGI Symbol;Acc:MGI:4439633] |
| -1.656 | 2.99E-03 | Iglc1 | IG_C_gene | immunoglobulin lambda constant 1 [Source:MGI Symbol;Acc:MGI:99546] |
| -6.182 | 3.01E-03 | Ighv2-9 | IG_V_gene | immunoglobulin heavy variable 2-9 [Source:MGI Symbol;Acc:MGI:4439624] |
| -6.302 | 3.01E-03 | Ighv2-5 | IG_V_gene | immunoglobulin heavy variable 2-5 [Source:MGI Symbol;Acc:MGI:4439517] |
| -2.399 | 3.19E-03 | Igkv10-96 | IG_V_gene | immunoglobulin kappa variable 10-96 [Source:MGI Symbol;Acc:MGI:4439561] |
| -4.608 | 5.00E-03 | Ighv1-50 | IG_V_gene | immunoglobulin heavy variable 1-50 [Source:MGI Symbol;Acc:MGI:4439753] |
| -6.025 | 5.10E-03 | Igkv4-80 | IG_V_gene | immunoglobulin kappa variable 4-80 [Source:MGI Symbol;Acc:MGI:4439653] |
| 2.253 | 5.41E-03 | Gm10591 | protein_coding | predicted gene 10591 [Source:MGI Symbol;Acc:MGI:3711256] |
| 2.144 | 5.93E-03 | Thbs4 | protein_coding | thrombospondin 4 [Source:MGI Symbol;Acc:MGI:1101779] |
| -5.075 | 6.61E-03 | Ighv5-6 | IG_V_gene | immunoglobulin heavy variable 5-6 [Source:MGI Symbol;Acc:MGI:4439815] |
| -1.415 | 7.10E-03 | Cd19 | protein_coding | CD19 antigen [Source:MGI Symbol;Acc:MGI:88319] |
| -2.556 | 9.51E-03 | Iglc3 | IG_C_gene | immunoglobulin lambda constant 3 [Source:MGI Symbol;Acc:MGI:99886] |
| -6.198 | 1.08E-02 | Ighv5-9-1 | IG_V_gene | immunoglobulin heavy variable 5-9-1 [Source:MGI Symbol;Acc:MGI:4439810] |
| -3.890 | 1.41E-02 | Igkv5-43 | IG_V_gene | immunoglobulin kappa chain variable 5-43 [Source:MGI Symbol;Acc:MGI:4943320] |
| -2.221 | 1.48E-02 | Ighv7-1 | IG_V_gene | immunoglobulin heavy variable 7-1 [Source:MGI Symbol;Acc:MGI:4439622] |
| -1.795 | 1.48E-02 | Ighd | IG_C_gene | immunoglobulin heavy constant delta [Source:MGI Symbol;Acc:MGI:96447] |
| -1.566 | 1.48E-02 | Fcmr | protein_coding | Fc fragment of IgM receptor [Source:MGI Symbol;Acc:MGI:1916419] |
| -2.699 | 1.55E-02 | Cr2 | protein_coding | complement receptor 2 [Source:MGI Symbol;Acc:MGI:88489] |
| -3.930 | 1.56E-02 | Ighv1-52 | IG_V_gene | immunoglobulin heavy variable 1-52 [Source:MGI Symbol;Acc:MGI:4439752] |
| -2.627 | 1.91E-02 | Igkv6-15 | IG_V_gene | immunoglobulin kappa variable 6-15 [Source:MGI Symbol;Acc:MGI:1330831] |
| -2.893 | 1.93E-02 | Rnase2a | protein_coding | ribonuclease, RNase A family, 2A (liver, eosinophil-derived neurotoxin) [Source:MGI Symbol;Acc:MGI:1890465] |
| -5.657 | 1.93E-02 | Ighv1-61 | IG_V_gene | immunoglobulin heavy variable 1-61 [Source:MGI Symbol;Acc:MGI:4439824] |
| -1.081 | 1.94E-02 | Fcrla | protein_coding | Fc receptor-like A [Source:MGI Symbol;Acc:MGI:2138647] |
| -3.414 | 2.01E-02 | Igkv12-44 | IG_V_gene | immunoglobulin kappa variable 12-44 [Source:MGI Symbol;Acc:MGI:4439775] |
| -1.785 | 2.70E-02 | Cacna1i | protein_coding | calcium channel, voltage-dependent, alpha 1I subunit [Source:MGI Symbol;Acc:MGI:2178051] |
| -4.348 | 2.96E-02 | Igkv8-19 | IG_V_gene | immunoglobulin kappa variable 8-19 [Source:MGI Symbol;Acc:MGI:1330844] |
| 1.452 | 2.97E-02 | Cxcl5 | protein_coding | chemokine (C-X-C motif) ligand 5 [Source:MGI Symbol;Acc:MGI:1096868] |
| -3.177 | 2.97E-02 | Ighv1-19 | IG_V_gene | immunoglobulin heavy variable V1-19 [Source:MGI Symbol;Acc:MGI:4439779] |
| -2.836 | 3.52E-02 | Igkv12-46 | IG_V_gene | immunoglobulin kappa variable 12-46 [Source:MGI Symbol;Acc:MGI:4439773] |
| -3.617 | 3.60E-02 | Ighv2-3 | IG_V_gene | immunoglobulin heavy variable 2-3 [Source:MGI Symbol;Acc:MGI:4439872] |
| -3.618 | 4.15E-02 | Igkv4-57 | IG_V_gene | immunoglobulin kappa variable 4-57 [Source:MGI Symbol;Acc:MGI:2685035] |
| -5.279 | 4.27E-02 | Igkv4-58 | IG_V_gene | immunoglobulin kappa variable 4-58 [Source:MGI Symbol;Acc:MGI:2685923] |
| -3.023 | 4.27E-02 | Igkv17-121 | IG_V_gene | immunoglobulin kappa variable 17-121 [Source:MGI Symbol;Acc:MGI:3647671] |
| -1.246 | 4.69E-02 | Ms4a1 | protein_coding | membrane-spanning 4-domains, subfamily A, member 1 [Source:MGI Symbol;Acc:MGI:88321] |
